# Supplementary material for: Foundations of a knee joint digital twin from qMRI biomarkers for osteoarthritis and knee replacement
Source: NPJ Digit Med. 2025 Feb 21;8:118. doi: 10.1038/s41746-025-01507-3 (PMC11845592; doi:10.1038/s41746-025-01507-3)
Supplement: Supplementary file 2 — Supplementary Information [file 41746_2025_1507_MOESM2_ESM.pdf]

Supplementary Information for: Foundations of a Knee Joint Digital Twin from qMRI Biomarkers for Osteoarthritis and Knee Replacement

Gabrielle Hoyer[1,2,3,*], Kenneth T Gao[1,2,3], Felix G Gassert[1], Johanna Luitjens[1], Fei Jiang[1], Sharmila Majumdar[1,2,3], Valentina Pedoia[1]

[1]Department of Radiology and Biomedical Imaging, University of California, San Francisco, San Francisco, CA, USA

[2]Department of Bioengineering, University of California Berkeley, Berkeley, CA, USA

[3]Department of Bioengineering, University of California San Francisco, San Francisco, CA, USA

* Correspondence: gabbie.hoyer@ucsf.edu

Table of Contents

Section 1: Supplementary Data Tables 5

Supplementary Data Alignment Note: 5

Supplementary Data 01: Variable Definitions for Cohort Study Analyses. 5

Supplementary Data 02: Baseline Demographic and Clinical Characteristics of Osteoarthritis Initiative Cohort 6

Supplementary Data 03: OAI Imaging Biomarkers 100-dimensional PCA Feature Space for Baseline Timepoint. 6

Supplementary Data 04: Kolmogorov-Smirnov Test Results for OA Incidence Sensitivity in Control vs. OA Incidence Groups (Before Matching). 7

Supplementary Data 05: Kolmogorov-Smirnov Test Results for Knee Replacement Sensitivity in Control vs. Knee Replacement Groups (Before Matching). 8

Supplementary Data 06: Composite Baseline Demographic and Clinical Characteristics of Osteoarthritis Initiative Cohort (Before and After Matching) 9

Supplementary Data 07: Composite Baseline Demographic and Clinical Characteristics of Knee Replacement Cohort (Before and After Matching) 9

Supplementary Data 08: Comparative Match Quality Indicators for OA Incidence Study Before and After Matching. 10

Supplementary Data 09: Normality and Homoscedasticity Assessment in OA Incidence Study (After Matching). 10

Supplementary Data 10: Comparative Match Quality Indicators for Knee Replacement Study Before and After Matching. 10

Supplementary Data 11: Normality and Homoscedasticity Assessment in Knee Replacement Study (After Matching). 10

Supplementary Data 12: Normality Evaluation for OA Incidence Clinical Cohort PC Modes After Matching. 11

Supplementary Data 13: Normality Evaluation for Knee Replacement Clinical Cohort PC Modes After Matching. 11

Supplementary Data 14: Adjusted Wilcoxon Rank Sum Test Results for OA Incidence Clinical Cohort PC Modes. 12

Supplementary Data 15: Adjusted Wilcoxon Rank Sum Test Results for Knee Replacement Clinical Cohort PC Modes. 12

Back to Table of Contents

Supplementary Data 16: Elastic Net Regularized GLM for Principal Component Mode Predictors in OA Incidence Study. 12

Supplementary Data 17: OA Incidence Predictive Feature Analysis via Bootstrap Elastic Net. 13

Supplementary Data 18: Final GLM Results for OA Incidence Study with Selected Features. 13

Supplementary Data 19: Elastic Net Regularized GLM for Principal Component Mode Predictors in Knee Replacement Study. 14

Supplementary Data 20: Knee Replacement Predictive Feature Analysis via Bootstrap Elastic Net. 14

Supplementary Data 21: Final GLM Results for Knee Replacement Study with Selected Features. 15

Supplementary Data 22: GLM Fit Statistics for OA Incidence Study. 16

Supplementary Data 23: GLM Fit Statistics for Knee Replacement Study. 16

Section 2: Supplementary Figures 17

Supplementary Figure 1: Variance Capture by Principal Components in Bone, Meniscus, and Cartilage Features. 17

Supplementary Figure 2: Top 10 Principal Component Modes for Femur Bone Shape Variability. 18

Supplementary Figure 3: Principal Component Analysis of Patella Bone Shape Variation. 19

Supplementary Figure 4: Variations in Tibia Bone Shape Across Principal Component Modes. 20

Supplementary Figure 5: Comparative Principal Component Analysis of Lateral and Medial Meniscus Shape Variations. 21

Supplementary Figure 6: Differential Cartilage Thickness in Femur Tissue Across Principal Component Extremes. 22

Supplementary Figure 7: Principal Component Analysis of Patella Cartilage Thickness Variation. 23

Supplementary Figure 8: Variability in Tibia Cartilage Thickness Through Principal Component Analysis. 24

Supplementary Figure 9: Principal Component Analysis of Femur Cartilage T_2 Relaxation Time Variability. 25

Supplementary Figure 10: Variations in Patella Cartilage T_2 Relaxation Times Across Principal Components. 26

Back to Table of Contents

Supplementary Figure 11: Principal Component Insights into Tibia Cartilage T_2 Relaxation Time Variability. 27

Supplementary Figure 12: Residual Distribution of Meniscus and Bone Shape Biomarkers in Knee Replacement Analysis 28

Supplementary Figure 13: Residual Distribution of Cartilage Thickness and T_2 Relaxation Biomarkers in Knee Replacement Analysis. 29

Supplementary Figure 14: Stability Selection of Imaging Biomarkers for OA Incidence and Knee Replacement Analyses. 30

Supplementary Figure 15: Histogram Distributions of Key Features in OA Incidence and Control Groups. 31

Supplementary Figure 16: Distribution Histograms of Key Predictive Features for Knee Replacement Risk. 32

Back to Table of Contents

Section 1: Supplementary Data Tables

Supplementary Data Alignment Note:

Tables that are too large to be included in this document can be found in the Supplementary_Data.xlsx file. In the event that any table presented inline within this document is difficult to read, we kindly recommend referring to the corresponding sheet in the Supplementary_Data.xlsx file for a clearer view. Each Excel sheet (S1, S2, S3, ..., S22) matches the numbering of the supplementary tables provided here and in the manuscript (Supplementary Table 1, 2, 3, ... 22).

Supplementary Data 01: Variable Definitions for Cohort Study Analyses.

This table serves as a detailed reference for variables utilized in the Cohort Study Analyses, with definitions drawn from the Osteoarthritis Initiative (OAI)[33] and KNOAP[54] Challenge datasets. It includes:

Demographic variables such as age, sex, and ethnicity.

Clinical variables encompassing measurements like height, weight, BMI, and various assessments of osteoarthritis severity.

The table systematically organizes variables by name, classification type, a description of their relevance to osteoarthritis research, and measurement details, providing a crucial resource for interpreting the analyses conducted in the study. Each variable is outlined in terms of its data capture method and the specific metrics used to ensure uniformity and precision across studies.

- See Supplementary_Data.xlsx for Table.

Back to Table of Contents

Supplementary Data 02: Baseline Demographic and Clinical Characteristics of Osteoarthritis Initiative Cohort

This table includes demographic and clinical characteristics from the Osteoarthritis Initiative cohort. It details the distribution of sex and race in both number and percentage forms. Additionally, it compares the median values for age, height, weight, body mass index (BMI), and clinical assessments derived from the WOMAC Index and the KOOS. The data are segmented into:

1) OAI Initial Subject Cohort - A general overview without group separation.

Osteoarthritis Initiative Baseline Demographics / Clinical Factors

Category	*n*	*%*
Sex Distribution		
Male	1789	41.77
Female	2494	58.23
Race Distribution		
Other non-White	67	1.56
White	3427	80.01
Black	751	17.53
Asian	38	0.89
	n	*Median [IQR]*
Continuous Demographic Measures		
Age (years)	4283	61 [16]
Height (mm)	4283	1675 [143.5]
Weight (kg)	4283	80.5 [22.3]
BMI	4283	28.2 [6.5]
WOMAC Index		
Pain	4283	1 [4]
ADL	4283	3 [12]
Stiffness	4283	1 [3]
Total	4283	6 [17]
KOOS		
Pain	4283	88.9 [25]
Symptom	4283	91.7 [21.4]
QOL	4283	68.8 [33.3]

Supplementary Data 03: OAI Imaging Biomarkers 100-dimensional PCA Feature Space for Baseline Timepoint.

Supplementary Data 03 presents the 100-dimensional PCA feature space for baseline imaging biomarkers from the OAI dataset, detailing the principal component modes analyzed for variations in bone shape, cartilage thickness, and T_2 relaxation time across key tissues, including the femur, patella, tibia, and menisci. These values form the foundation for the statistical shape modeling and dimensionality reduction processes described in the study.

- See Supplementary_Data.xlsx for Table.

Back to Table of Contents

Supplementary Data 04: Kolmogorov-Smirnov Test Results for OA Incidence Sensitivity in Control vs. OA Incidence Groups (Before Matching).

Supplementary Data 04 presents the results of the Kolmogorov-Smirnov tests conducted to assess the sensitivity of multiple imputation in the control and osteoarthritis (OA) incidence groups prior to matching. It details the distributions' comparison for key variables to determine the similarity between the imputed and observed data, guiding the imputation's validity.

OA Incidence (Before Matching)<br>Kolmogorov-Smirnov Tests - OA Incidence Sensitivity Test for Multiple Imputation				
Category	*Control Group*		*OA Incidence Group*	
	KS Statistic	*P-Value*	*KS Statistic*	*P-Value*
Hispanic	6.12E-06	1.00E+00	0.00E+00	1.00E+00
Race	2.82E-04	1.00E+00	9.76E-05	1.00E+00
Gender	0.00E+00	1.00E+00	0.00E+00	1.00E+00
Tenderness	9.84E-03	1.00E+00	4.67E-04	1.00E+00
Injury History	2.51E-03	1.00E+00	2.49E-03	1.00E+00
Mild Symptoms	1.99E-04	1.00E+00	1.80E-04	1.00E+00
Heberden	3.10E-04	1.00E+00	1.64E-04	1.00E+00
Crepitus	1.18E-02	9.99E-01	3.95E-04	1.00E+00
Morning Stiffness	7.03E-05	1.00E+00	3.55E-04	1.00E+00
Continuous Demographic Measures				
Age (years)	0.00E+00	1.00E+00	0.00E+00	1.00E+00
Height (mm)	9.06E-03	1.00E+00	8.82E-03	1.00E+00
Weight (kg)	0.00E+00	1.00E+00	6.97E-04	1.00E+00
BMI	0.00E+00	1.00E+00	9.59E-04	1.00E+00
WOMAC Index				
Pain	3.67E-04	1.00E+00	0.00E+00	1.00E+00
ADL	5.83E-04	1.00E+00	2.05E-03	1.00E+00
Stiffness	0.00E+00	1.00E+00	3.11E-04	1.00E+00
Total	1.09E-03	1.00E+00	2.03E-03	1.00E+00
KOOS				
Pain	3.75E-04	1.00E+00	0.00E+00	1.00E+00
Symptom	0.00E+00	1.00E+00	0.00E+00	1.00E+00
QOL	2.81E-04	1.00E+00	0.00E+00	1.00E+00

Back to Table of Contents

Supplementary Data 05: Kolmogorov-Smirnov Test Results for Knee Replacement Sensitivity in Control vs. Knee Replacement Groups (Before Matching).

Supplementary Data 05 displays the outcomes of the Kolmogorov-Smirnov tests applied to evaluate the sensitivity of multiple imputation for the control group versus the knee replacement group before matching. It compares distributions of essential variables, scrutinizing the alignment between imputed values and actual data to ensure accurate imputation.

Knee Replacement (Before Matching)

Kolmogorov-Smirnov Tests - Knee Replacement Sensitivity Test for Multiple Imputation

Category	*Control Group*		*Knee Replacement Group*	
	KS Statistic	*P-Value*	*KS Statistic*	*P-Value*
Hispanic	2.77E-06	1.00E+00	0.00E+00	1.00E+00
Race	6.13E-05	1.00E+00	0.00E+00	1.00E+00
Gender	0.00E+00	1.00E+00	0.00E+00	1.00E+00
Tenderness	7.17E-03	1.00E+00	1.63E-03	1.00E+00
Injury History	2.80E-03	1.00E+00	4.63E-03	1.00E+00
Mild Symptoms	7.45E-05	1.00E+00	0.00E+00	1.00E+00
Heberden	6.19E-04	1.00E+00	1.62E-03	1.00E+00
Crepitus	8.43E-03	9.99E-01	1.49E-03	1.00E+00
Morning Stiffness	1.93E-04	1.00E+00	0.00E+00	1.00E+00
Continuous Demographic Measures				
Age (years)	0.00E+00	1.00E+00	0.00E+00	1.00E+00
Height (mm)	8.32E-03	9.99E-01	1.06E-02	1.00E+00
Weight (kg)	6.39E-04	1.00E+00	0.00E+00	1.00E+00
BMI	4.81E-04	1.00E+00	0.00E+00	1.00E+00
WOMAC Index				
Pain	1.41E-04	1.00E+00	0.00E+00	1.00E+00
ADL	1.23E-03	1.00E+00	2.15E-03	1.00E+00
Stiffness	1.52E-04	1.00E+00	0.00E+00	1.00E+00
Total	1.52E-03	1.00E+00	2.27E-03	1.00E+00
KOOS				
Pain	1.56E-04	1.00E+00	0.00E+00	1.00E+00
Symptom	0.00E+00	1.00E+00	0.00E+00	1.00E+00
QOL	1.61E-04	1.00E+00	0.00E+00	1.00E+00

Back to Table of Contents

Supplementary Data 06: Composite Baseline Demographic and Clinical Characteristics of Osteoarthritis Initiative Cohort (Before and After Matching)

This table combines four different analyses of demographic and clinical characteristics from the Osteoarthritis Initiative cohort, differentiated by OA incidence and control groups, and by the matching process applied. It details the distribution of sex and race in both number and percentage forms. Additionally, it compares the median values for age, height, weight, body mass index (BMI), and clinical assessments derived from the WOMAC Index and the KOOS. The data are segmented into:

1) OA Incidence (Before Matching) - Separated into non-OA / OA Incidence subjects (progressing at any timepoint)

2) OA Incidence (Before Matching) - Focused on subjects with OA incidence post-baseline, non-OA at baseline, split by Control/OA Incidence groups.

3) OA Incidence (After Matching) - Post-matching demographics and clinical factors compared between Control and OA Incidence groups.

Each segment presents counts and percentages for categorical measures and medians with interquartile ranges for continuous variables, facilitating a comprehensive comparison across the cohort's subgroups both before and after the matching process.

- See Supplementary_Data.xlsx for Table.

Supplementary Data 07: Composite Baseline Demographic and Clinical Characteristics of Knee Replacement Cohort (Before and After Matching)

This table consolidates three separate analyses of demographic and clinical data from the Knee Replacement Cohort within the Osteoarthritis Initiative, categorized by individuals who underwent knee replacement (TKR) and corresponding control groups, as well as by the application of matching criteria. It systematically details sex and race distributions in numerical counts and percentages and compares median values for age, height, weight, BMI, and clinical evaluations based on the WOMAC Index and the KOOS. The dataset is arranged into:

1) TKR (Before Matching) - Separates data into Control and Knee Replacement groups to facilitate a before-matching comparison.

2) TKR (After Matching) - Offers a demographic and clinical juxtaposition between Control and Knee Replacement groups following the matching process.

Each part of the table presents categorical information in terms of frequencies and proportions, and continuous variables as medians accompanied by interquartile ranges. This format allows for an in-depth look at the foundational attributes of the cohorts both before and after matching, underscoring the pertinent demographic and clinical factors relevant to knee replacement studies.

- See Supplementary_Data.xlsx for Table.

Back to Table of Contents

Supplementary Data 08: Comparative Match Quality Indicators for OA Incidence Study Before and After Matching.

Supplementary Data 08 illustrates the match quality for the OA Incidence Study through Cramer's V for categorical variables, and Cohen's d along with the Point Biserial Correlation Coefficient for continuous variables. The table displays how these values change from before to after matching, reflecting the matching procedure's influence on the equivalence of the study's groups in terms of demographic and clinical variables.

- See Supplementary_Data.xlsx for Table.

Supplementary Data 09: Normality and Homoscedasticity Assessment in OA Incidence Study (After Matching).

Supplementary Data 09 assesses the distribution and variance of covariates in the OA Incidence Study after matching. It includes results from both the Shapiro-Wilk and Anderson-Darling test for normality and examines homoscedasticity to confirm that the variances are equal across groups. Additionally, the table reports on Paired Wilcoxon Rank Sum Tests and Chi-Squared Tests, indicating whether covariates maintain their expected distributions post-matching. The significance levels for the Anderson-Darling test are given, and if the test statistic is above these values, the assumption of normality at the respective significance levels is not met.
Significance levels for the Anderson-Darling test: 0.57 (15%), 0.649 (10%), 0.778 (5%), 0.908 (2.5%), 1.08 (1%).

- See Supplementary_Data.xlsx for Table.

Supplementary Data 10: Comparative Match Quality Indicators for Knee Replacement Study Before and After Matching.

Supplementary Data 10 provides the match quality evaluation for the Knee Replacement Study using Cramer's V for categorical variables, and Cohen's d together with the Point Biserial Correlation Coefficient for continuous variables. It contrasts these indicators before and after the matching process, demonstrating the degree to which the matching has adjusted the group characteristics to reduce bias in the comparative analyses.

- See Supplementary_Data.xlsx for Table.

Supplementary Data 11: Normality and Homoscedasticity Assessment in Knee Replacement Study (After Matching).

Supplementary Data 11 provides an evaluation of normality and variance consistency for covariates in the Knee Replacement Study after the matching procedure. This includes results from both the Shapiro-Wilk and Anderson-Darling tests for normality and assessments of homoscedasticity, as well as outcomes from Paired Wilcoxon Rank Sum Tests and Chi-Squared Tests. The table delineates critical values for the Anderson-Darling test at various significance levels. Test statistics surpassing these thresholds indicate a

deviation from the assumed distribution at the corresponding levels of significance. Significance levels for the Anderson-Darling test: 0.567 (15%), 0.646 (10%), 0.775 (5%), 0.904 (2.5%), 1.075 (1%).

- See Supplementary_Data.xlsx for Table.

Supplementary Data 12: Normality Evaluation for OA Incidence Clinical Cohort PC Modes After Matching.

Supplementary Data 12 presents a rigorous evaluation of the normality and equal variance of principal component (PC) modes related to bone shape, meniscus shape, cartilage thickness, and cartilage T_2 relaxation in the OA Incidence Clinical Cohort study, post-matching. The Shapiro-Wilk test results are documented through the W statistic and P-value, whereas the Anderson-Darling test outcomes are detailed with the test statistic and its significance level. Critical values for the latter are specified, and exceeding these indicates a departure from the assumed normal distribution at the stated significance levels.

Significance levels for the Anderson-Darling test: 0.57 (15%), 0.649 (10%), 0.778 (5%), 0.908 (2.5%), 1.08 (1%).

Footnote - departure from normality indicative of significant p-value:

*p < 0.05

**p < 0.01

***p < 0.001

- See Supplementary_Data.xlsx for Table.

Supplementary Data 13: Normality Evaluation for Knee Replacement Clinical Cohort PC Modes After Matching.

Supplementary Data 13 offers an in-depth analysis of normality and variance homogeneity for PC modes concerning bone shape, meniscus shape, cartilage thickness, and cartilage T_2 relaxation in the Knee Replacement Clinical Cohort study, following the matching process. The table provides Shapiro-Wilk test W statistics and P-values, along with Anderson-Darling test statistics and significance levels. The specified critical values for the Anderson-Darling test serve as thresholds, with values beyond these suggesting non-adherence to the presupposed distribution at the respective significance levels. Significance levels for the Anderson-Darling test: 0.567 (15%), 0.646 (10%), 0.775 (5%), 0.904 (2.5%), 1.075 (1%).

Footnote - departure from normality indicative of significant p-value:

*p < 0.05

**p < 0.01

***p < 0.001

- See Supplementary_Data.xlsx for Table.

Back to Table of Contents

Supplementary Data 14: Adjusted Wilcoxon Rank Sum Test Results for OA Incidence Clinical Cohort PC Modes.

Supplementary Data 14 consolidates the results of Benjamini-Hochberg corrected Paired Wilcoxon Rank Sum Tests for principal component modes in the OA Incidence Clinical Cohort study. It includes categories, Wilcoxon statistics, raw and adjusted P-values, degrees of freedom (df), and confidence intervals (CIs) at both 95% and 99% for point estimates. The footnote indicates the significance levels for interpreting the P-values:

*p < 0.05

**p < 0.01

***p < 0.001

- See Supplementary_Data.xlsx for Table.

Supplementary Data 15: Adjusted Wilcoxon Rank Sum Test Results for Knee Replacement Clinical Cohort PC Modes.

Supplementary Data 15 details the Benjamini-Hochberg corrected Paired Wilcoxon Rank Sum Tests applied to the principal component modes in the KR Clinical Cohort study. It tabulates the same columns as S14, providing a statistical overview that includes the Wilcoxon-stat, unadjusted and adjusted P-values, df, and both 95% and 99% confidence intervals for point estimates. The table's footnote communicates the significance thresholds for the P-values:

*p < 0.05

**p < 0.01

***p < 0.001

- See Supplementary_Data.xlsx for Table.

Supplementary Data 16: Elastic Net Regularized GLM for Principal Component Mode Predictors in OA Incidence Study.

Supplementary Data 16 displays results from applying elastic net regularization within a generalized linear model (GLM) to predict control and OA incidence outcomes using principal component modes and covariates. Iterations over 1,000 bootstrap samples are documented, highlighting the variables most contributive to model decision-making across iterations.

- See Supplementary_Data.xlsx for Table.

Back to Table of Contents

Supplementary Data 17: OA Incidence Predictive Feature Analysis via Bootstrap Elastic Net.

Supplementary Data 17 provides an analysis of features from the OA Incidence Study using a bootstrap elastic net approach with 1,000 iterations. It details each feature's mean coefficient, standard deviation, absolute mean coefficient, 95% and 99% confidence intervals, and the frequency of selection. Additionally, the weighted importance and rank of each feature are listed, along with indicators for inclusion within the 95% and 99% confidence intervals. The table is intended to elucidate the stable and significant contributors to the model's predictive capacity for the OA incidence outcome.

- See Supplementary_Data.xlsx for Table.

Supplementary Data 18: Final GLM Results for OA Incidence Study with Selected Features.

Supplementary Data 18 encapsulates the outcomes of the generalized linear model (GLM) applied to the OA Incidence Study, incorporating only those features whose weighted importance ranked in the upper quartile. The table presents each selected feature's logistic regression coefficient, standard error, P-value, confidence interval, and odds ratio. These parameters indicate the significance and strength of the association each feature has with the incidence of osteoarthritis, after refinement through stability selection.

OA Incidence Multivariate Regression - GLM + Selected Features Model Results

Variable	*CI 2.5%*	*CI 97.5%*	*Odds Ratio*	*p-value*	*Std.Err.*	*z-value*
const	0.064	5.871	0.615	0.672514	1.151	-0.423
Age	0.969	1.012	0.990	0.378992	0.011	-0.880
BMI	0.990	1.071	1.030	0.145813	0.020	1.454
Sex	0.707	2.116	1.223	0.471782	0.280	0.720
Race	0.809	1.981	1.266	0.301618	0.228	1.033
Crepitus	0.543	1.097	0.772	0.148620	0.179	-1.444
Cartilage T2 Femur PC 6	0.953	1.002	0.977	0.077217	0.013	-1.767
Cartilage T2 Femur PC 7	0.918	0.982	0.949	0.002355	0.017	-3.041
Cartilage T2 Patella PC 3	1.001	1.099	1.049	0.043723	0.024	2.017
Cartilage T2 Patella PC 4	0.902	0.999	0.950	0.046773	0.026	-1.988
Cartilage T2 Patella PC 6	0.916	1.027	0.970	0.299068	0.029	-1.038
Cartilage T2 Patella PC 8	0.894	1.020	0.955	0.173802	0.034	-1.360
Cartilage T2 Patella PC 10	0.966	1.128	1.044	0.279063	0.039	1.082
Cartilage T2 Tibia PC 5	0.991	1.083	1.036	0.114570	0.023	1.578
Cartilage T2 Tibia PC 6	0.983	1.075	1.028	0.231591	0.023	1.196
Cartilage T2 Tibia PC 7	0.888	0.985	0.935	0.011047	0.026	-2.541
Cartilage T2 Tibia PC 8	0.898	0.992	0.944	0.023140	0.025	-2.271
Cartilage T2 Tibia PC 9	0.920	1.021	0.969	0.234985	0.027	-1.188
Cartilage Thickness Femur PC 1	0.959	0.987	0.973	0.000182	0.007	-3.742
Cartilage Thickness Femur PC 2	0.935	0.985	0.960	0.002051	0.013	-3.083
Cartilage Thickness Femur PC 6	0.993	1.053	1.023	0.129453	0.015	1.516
Cartilage Thickness Femur PC 10	0.951	1.016	0.983	0.304014	0.017	-1.028
Cartilage Thickness Patella PC 1	1.005	1.049	1.027	0.016192	0.011	2.405
Cartilage Thickness Patella PC 2	0.936	1.006	0.971	0.107293	0.019	-1.610
Cartilage Thickness Patella PC 6	0.901	1.001	0.950	0.054838	0.027	-1.920
Cartilage Thickness Patella PC 7	1.004	1.121	1.061	0.036096	0.028	2.096
Cartilage Thickness Patella PC 10	0.956	1.106	1.028	0.457217	0.037	0.743
Cartilage Thickness Tibia PC 1	0.999	1.045	1.022	0.058316	0.011	1.893
Cartilage Thickness Tibia PC 5	1.002	1.079	1.039	0.040966	0.019	2.044
Cartilage Thickness Tibia PC 7	0.903	0.984	0.943	0.007444	0.022	-2.676
Cartilage Thickness Tibia PC 8	0.988	1.079	1.033	0.148905	0.022	1.443
Cartilage Thickness Tibia PC 9	0.907	1.003	0.954	0.063686	0.025	-1.854
Medial Meniscus PC 10	1.002	1.051	1.026	0.032845	0.012	2.134
Lateral Meniscus PC 4	0.992	1.005	0.998	0.639808	0.003	-0.468
Lateral Meniscus PC 8	0.980	1.013	0.996	0.632327	0.008	-0.478
Lateral Meniscus PC 9	0.955	1.001	0.978	0.062101	0.012	-1.866

Back to Table of Contents

Supplementary Data 19: Elastic Net Regularized GLM for Principal Component Mode Predictors in Knee Replacement Study.

Supplementary Data 19 outlines the outcomes of elastic net regularization coupled with a generalized linear model (GLM) for identifying predictive principal component modes and covariates in distinguishing between control and knee replacement groups. Each row represents an iteration within the 1,000 bootstrap samples, revealing the predictive value of each variable.

- See Supplementary_Data.xlsx for Table.

Supplementary Data 20: Knee Replacement Predictive Feature Analysis via Bootstrap Elastic Net.

Supplementary Data 20 outlines the results of a bootstrap elastic net model for the Knee Replacement Study, iterating over 1,000 samples. Similar to Supplementary Data 17, it includes statistics for each feature's mean coefficient, variability, absolute impact, confidence intervals at 95% and 99%, and the stability of selection across bootstraps. The derived weighted importance score helps to highlight the features that consistently affect the model's predictive accuracy for knee replacement outcomes. Features with the highest weighted importance, representing the top quartile, are deemed the most robust for inclusion in the model.

- See Supplementary_Data.xlsx for Table.

Back to Table of Contents

Supplementary Data 21: Final GLM Results for Knee Replacement Study with Selected Features.

Supplementary Data 21 displays the final logistic regression results of the GLM for the Knee Replacement Study, post-feature selection. It details the logistic regression coefficients, standard errors, P-values, confidence intervals, and odds ratios for the features with the highest weighted importance. This final model assessment reveals the selected features' influence on the likelihood of knee replacement, with a focus on the statistical significance and effect sizes demonstrated by the refined predictor set.

Knee Replacement Multivariate Regression - GLM + Selected Features Model Results

Variable	*CI 2.5%*	*CI 97.5%*	*Odds Ratio*	*p-value*	*Std.Err.*	*z-value*
const	0.085	26.110	1.490	0.784774	1.461	0.273
Race	0.344	1.015	0.591	0.056808	0.276	-1.905
Crepitus	0.528	1.387	0.856	0.527548	0.246	-0.632
Age	0.944	1.002	0.973	0.066705	0.015	-1.834
Sex	0.640	2.052	1.146	0.645530	0.297	0.460
BMI	0.953	1.052	1.001	0.966358	0.025	0.042
KOOS Symptom	0.999	1.025	1.012	0.060957	0.007	1.874
Bone Shape Tibia PC 9	1.016	1.059	1.038	0.000482	0.011	3.490
Cartilage T2 Femur PC 2	0.971	1.010	0.991	0.348607	0.010	-0.937
Cartilage T2 Femur PC 5	1.027	1.102	1.064	0.000611	0.018	3.427
Cartilage T2 Femur PC 10	1.026	1.122	1.073	0.002038	0.023	3.085
Cartilage T2 Patella PC 1	0.971	1.018	0.995	0.647805	0.012	-0.457
Cartilage T2 Patella PC 5	0.984	1.122	1.051	0.137340	0.033	1.486
Cartilage T2 Patella PC 6	0.962	1.117	1.036	0.347880	0.038	0.939
Cartilage T2 Patella PC 7	0.862	1.003	0.930	0.058792	0.039	-1.890
Cartilage T2 Patella PC 9	1.041	1.259	1.145	0.005309	0.048	2.788
Cartilage T2 Patella PC 10	0.932	1.136	1.029	0.570961	0.051	0.567
Cartilage T2 Tibia PC 2	0.938	1.009	0.973	0.133175	0.019	-1.502
Cartilage T2 Tibia PC 7	0.833	0.952	0.890	0.000618	0.034	-3.424
Cartilage T2 Tibia PC 8	0.937	1.059	0.996	0.906197	0.031	-0.118
Cartilage T2 Tibia PC 10	0.995	1.127	1.059	0.071331	0.032	1.803
Cartilage Thickness Femur PC 2	0.927	0.970	0.948	0.000004	0.012	-4.618
Cartilage Thickness Femur PC 9	0.962	1.040	1.000	0.989602	0.020	0.013
Cartilage Thickness Patella PC 3	1.015	1.110	1.062	0.008692	0.023	2.624
Cartilage Thickness Patella PC 7	0.929	1.065	0.995	0.877708	0.035	-0.154
Cartilage Thickness Patella PC 8	0.873	1.030	0.948	0.208033	0.042	-1.259
Cartilage Thickness Patella PC 9	0.954	1.136	1.041	0.362100	0.044	0.911
Cartilage Thickness Tibia PC 1	1.014	1.054	1.034	0.000752	0.010	3.370
Cartilage Thickness Tibia PC 2	0.911	0.968	0.939	0.000046	0.015	-4.076
Cartilage Thickness Tibia PC 3	0.983	1.047	1.015	0.363528	0.016	0.909
Cartilage Thickness Tibia PC 4	0.934	1.009	0.971	0.131752	0.020	-1.507
Cartilage Thickness Tibia PC 5	1.012	1.111	1.060	0.014079	0.024	2.455
Cartilage Thickness Tibia PC 7	0.974	1.072	1.022	0.372789	0.024	0.891
Cartilage Thickness Tibia PC 8	0.884	0.983	0.932	0.009397	0.027	-2.597
Cartilage Thickness Tibia PC 10	0.914	1.032	0.971	0.348002	0.031	-0.938

Back to Table of Contents

Supplementary Data 22: GLM Fit Statistics for OA Incidence Study.

Supplementary Data 22 provides key fit statistics for the GLM applied to the OA Incidence Study, including AIC, BIC, log-likelihood, Pearson Chi-Square, and Pseudo R-squared.

OA Incidence Regression Statistics	
AIC	925.8918585
BIC	-3601.16679
BIC LLF	1090.443645
LLF	-426.9459293
Pearson chi2	715.0585854
Pseudo R-squared	0.173344625

Supplementary Data 23: GLM Fit Statistics for Knee Replacement Study.

Supplementary Data 23 lists the fit statistics for the GLM used in the Knee Replacement Study, featuring AIC, BIC, log-likelihood, Pearson Chi-Square, and Pseudo R-squared values.

Knee Replacement Regression Statistics	
AIC	593.0783519
BIC	-2409.620419
BIC LLF	741.0071353
LLF	-261.539176
Pearson chi2	568.8086769
Pseudo R-squared	0.297101429

Back to Table of Contents

Section 2: Supplementary Figures

Supplementary Figure 1: Variance Capture by Principal Components in Bone, Meniscus, and Cartilage Features.

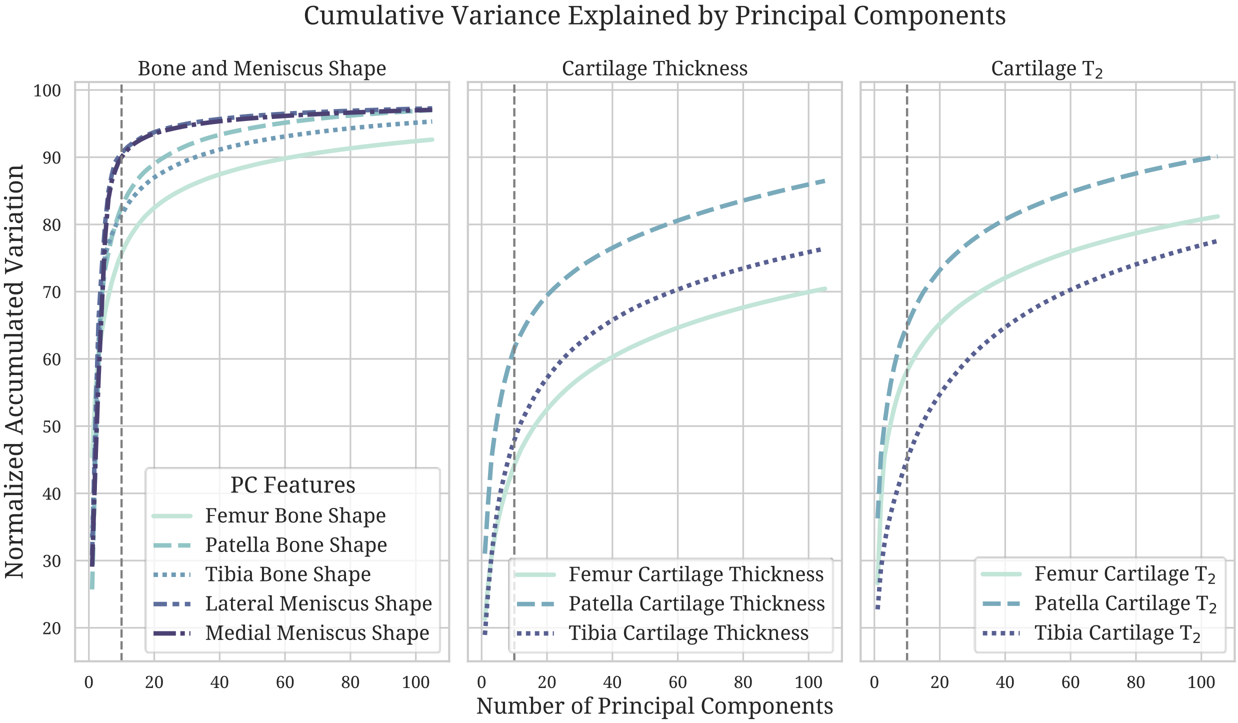

Bone shape modes (Femur, Patella, and Tibia) and Meniscus shape modes (Lateral and Medial) collectively demonstrate a high level of compactness, with each exceeding 80% variance captured at the 10 principal component threshold. Cartilage Thickness modes (Femur, Patella, and Tibia) and Cartilage T_2 modes (Femur, Patella, and Tibia) display a compactness capturing approximately 45-65% of the variance at the same 10 mode principal component mark.

Back to Table of Contents

Supplementary Figure 2: Top 10 Principal Component Modes for Femur Bone Shape Variability.

PC	-3 STD **Bone Shape** +3 STD	Interpretation
1	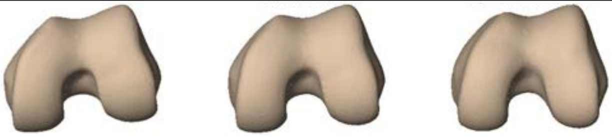	Widening of the left condyle, shortening (anterior to posterior) of both condyles
2	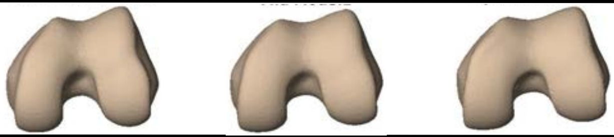	Thinning of the right condyle, indentation of the left articular surface
3	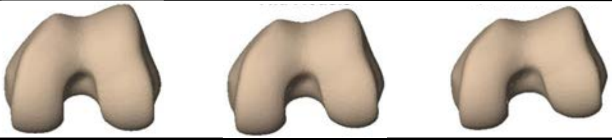	Enlargement (anterior to posterior) of the left condyle
4	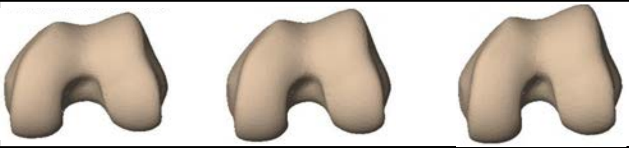	Shortening (anterior to posterior) of both condyles, widening of the intercondylar notch
5	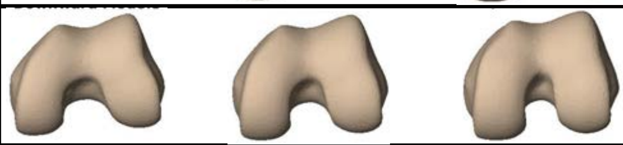	Shortening (anterior to posterior) of both condyles, Strong widening of the intercondylar notch, indentation in the left articular surface with kinking to the lateral
6	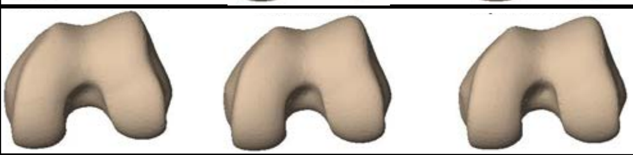	Widening of the intercodylar notch, more prominent right condyle
7	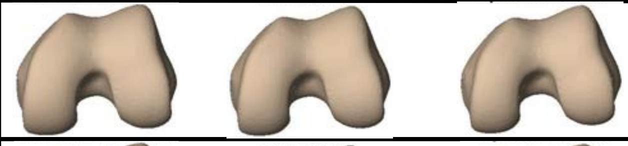	Slight thinning of the intercondylar notch, slight thickening of the right condyle
8	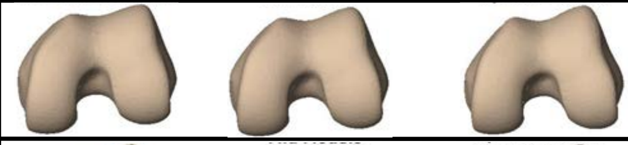	Thickening of the left condyle, lower angle in the trochlear surface
9	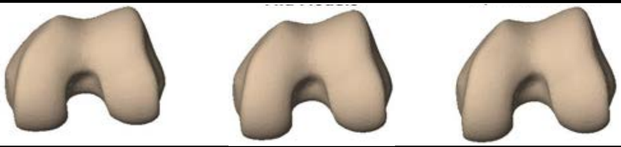	Thinning of the right condyle, lower angle of the trochlea surface
10	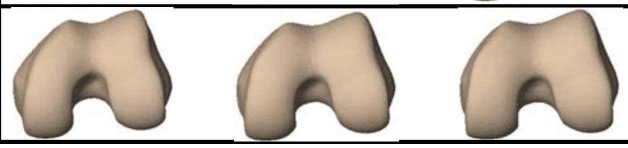	Slight shortening of the left condyle and more prominent of the right condyle

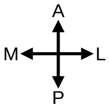

This visual table elucidates the spectrum of Femur bone shape variations within the OAI[33] patient population, presented through a series of 3D models. Each row represents one of the top 10 principal components (PCs), displaying the shape variation at -3 standard deviations (left), the mean mode (center), and +3 standard deviations (right). Accompanying interpretations (presented for -3 standard deviation) by radiologist experts provide insights into the anatomical significance of each PC mode, offering a detailed perspective on the morphological diversity associated with the femur across individuals. This figure includes an orientation legend to clarify anatomical directions: A (anterior), L (lateral), P (posterior), and M (medial), which are consistent across subsequent figures.

Back to Table of Contents

Supplementary Figure 3: Principal Component Analysis of Patella Bone Shape Variation.

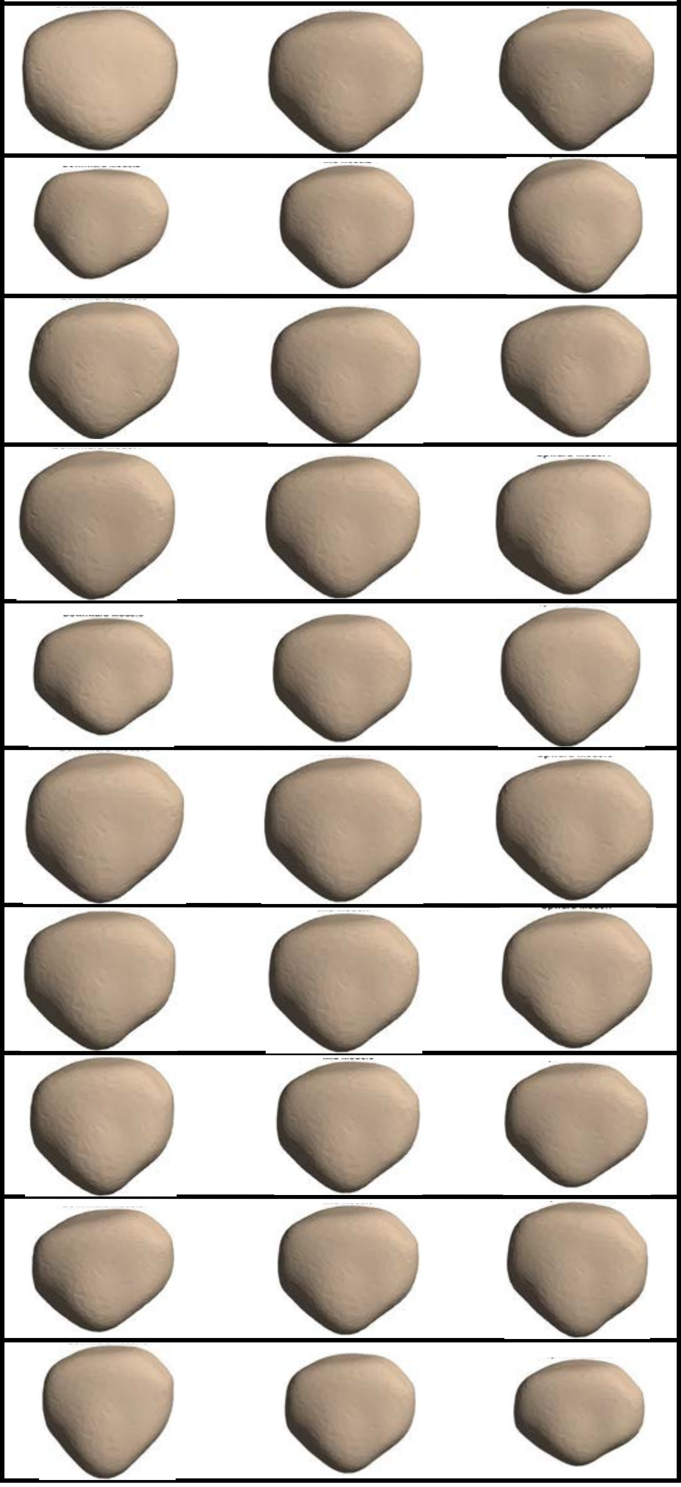

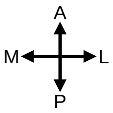

PC	-3 STD	Bone Shape	+3 STD	Interpretation
1				Rounding of apex, Flattening of anterior surface
2				Rotation counterclockwise, Apex tilted to the left, shortening (proximal to distal)
3				Shift of vertical ridge to the left, rotation counterclockwise, more prominent indentation of right articular surface
4				Elongation (proximal to distal), more prominent apex, rounding of the base, slightly more prominent vertical ridge
5				Shortening, more square-like shape with flattened apex, more prominent vertical ridge
6				Enlargement (proximal to distal)and rounding of entire patellar, tilting to the left
7				Indentation of the right articular surface, slightly more pointy apex
8				Enlargement (proximal to distal) and rounding of the base, flattening of right articular surface
9				Shortening (proximal to distal), Shift of vertical ridge to the left, steeper declension of the left facet, less rounding of the left button corner
10				Enlargement (proximal to distal), bigger apex

Displayed within this figure are 3D models capturing the variability in Patella bone shape across the OAI[33] patient population, aligned with the top 10 principal components (PCs). Each row visualizes the bone shape alteration at -3 standard deviations (left), the mean model (center), and +3 standard deviations (right). Expert radiological interpretation interpretations (presented for -3 standard deviation) is provided to elucidate the clinical relevance of the deviations, thus revealing the intricate morphological differences manifested in the patella bone structure.

Back to Table of Contents

Supplementary Figure 4: Variations in Tibia Bone Shape Across Principal Component Modes.

PC	-3 STD **Bone Shape** +3 STD	Interpretation
1	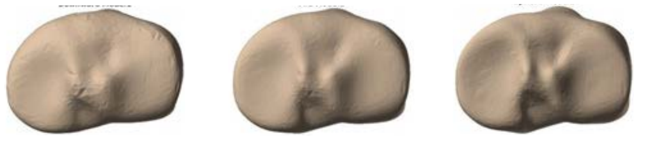	Flattening of articular surface on both sides
2	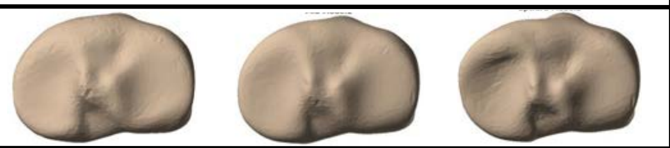	Flattening of the right articular surface, rounding of the entire joint surface
3	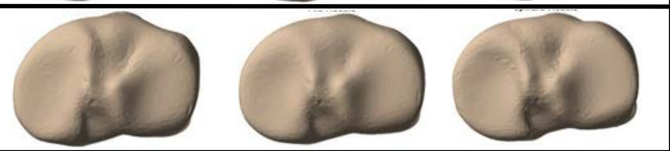	Enlargement of the right surface to anterior, slight elevation of the anterior part of left surface, more square-like shape
4	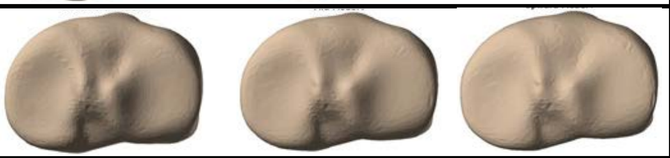	Enlargement of the anterior part of the left articular surface
5	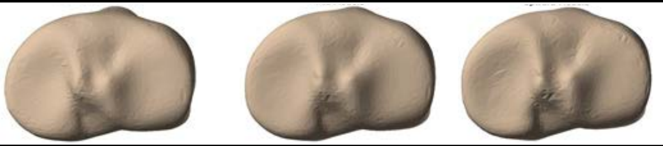	Flattening of the right articular surface, enlargement of the anterior part of the left surface
6	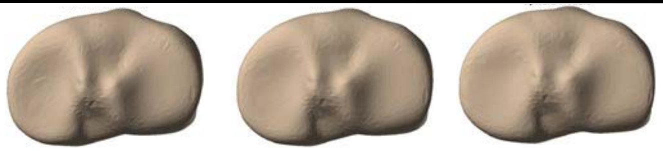	Stronger indentation of the left articular surface
7	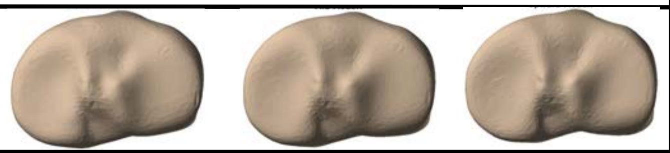	Slight clockwise rotation of the surface
8	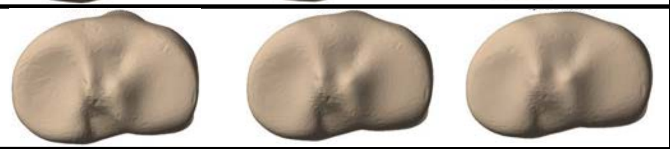	Stronger indentation of the right articular surface with areal increase in the right anterior part, more prominent right intercondylar tubercle
9	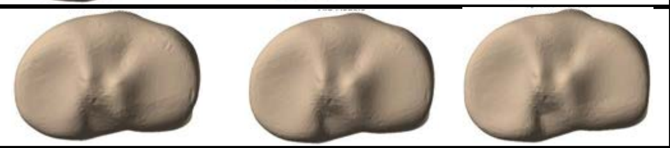	More prominent intercondylar tubercle, slight loss in the anterior part of the right surface
10	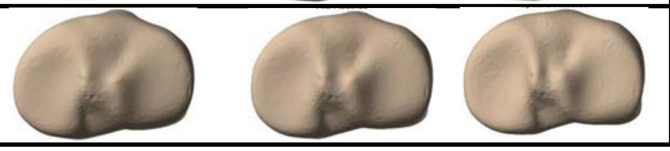	More rounding of the left articular surface, slight flattening of the right articular surface

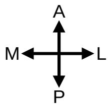

This extended figure presents a detailed examination of the Tibia bone shape diversity within the study's population, visualized through the lens of the top 10 principal components (PCs). Each row provides a comparative display of the bone shape at -3 standard deviations (left), the average bone shape (center), and +3 standard deviations (right). Annotations from radiological experts, focused on the -3 standard deviation models, offer interpretative insights into the significant morphological variations that are characteristic of the tibia bone at these deviations.

Back to Table of Contents

Supplementary Figure 5: Comparative Principal Component Analysis of Lateral and Medial Meniscus Shape Variations.

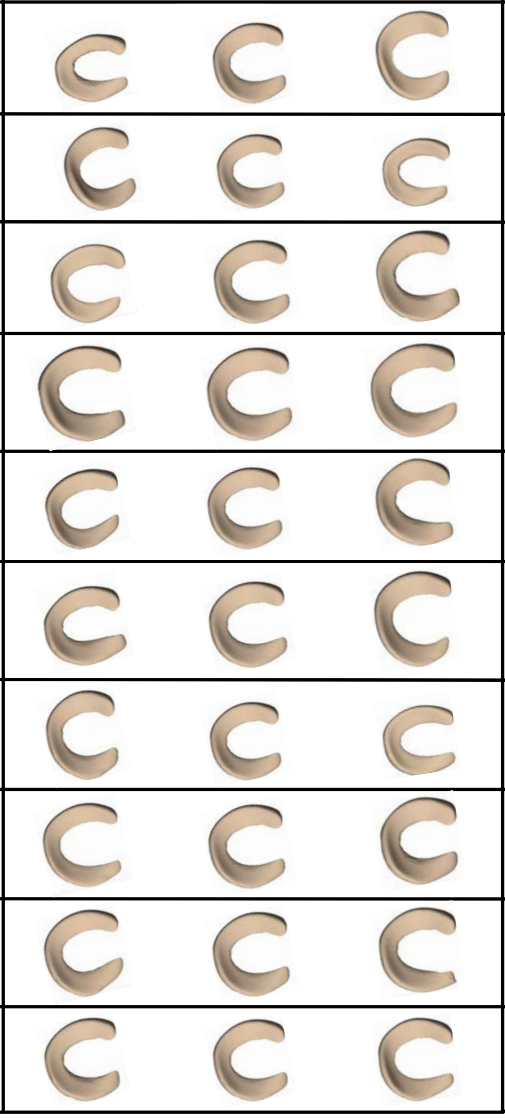

PC	Lateral Shape (-3 STD … +3 STD)	Interpretation
1		Overall smaller arch with thicker and smaller anterior and posterior horn as well as wider body; overall wider arch with thinner and wider anterior and posterior horn as well as thicker and smaller body.
2		Overall wider arch with thicker main body; overall smaller arch with thinner main body.
3		Overall smaller arch with a more tapered anterior horn; slight widening and thinning of the anterior horn.
4		Slightly thicker main body with wider anterior horn; slightly thinner main body with slightly more tapered anterior horn
5		Smaller and more posterior pointing anterior horn; wider and more anterior pointing anterior horn.
6		Shorter main body with longer and flatter anterior horn; longer main body with shorter and more hook-shaped anterior horn.
7		Overall wider arch with wider and slightly shorter anterior and posterior horn; overall smaller arch with slightly thinner and longer anterior and posterior horn.
8		Overall smaller and thinner entire meniscus; overall wider and thicker entire meniscus.
9		More posterior facing and tapered anterior horn; more posterior facing and blunt anterior horn.
10		Slightly more blunt anterior horn and thinner main body; slightly more tapered anterior horn and thicker main body.

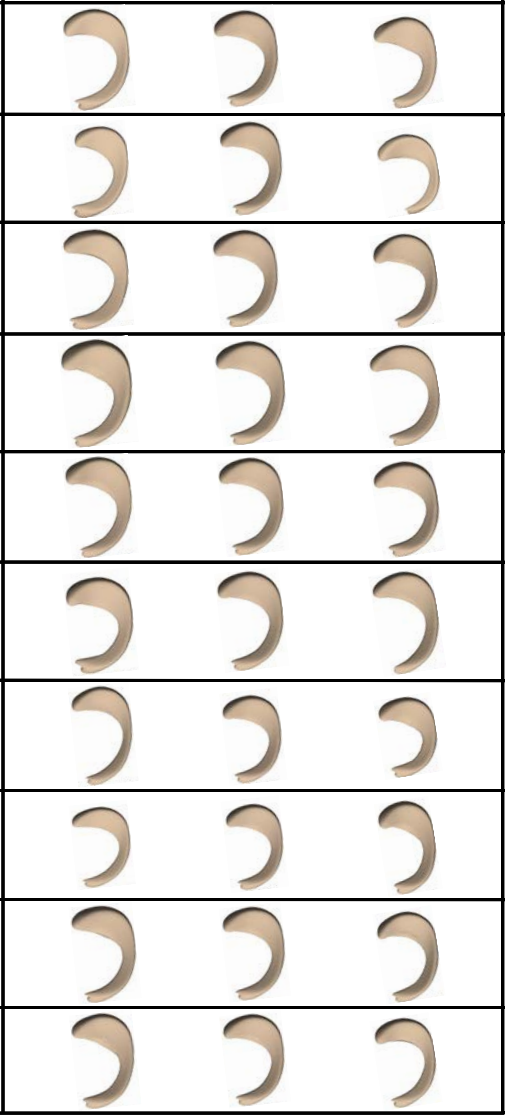

PC	Medial Shape (-3 STD … +3 STD)	Interpretation
1		Wider arch with thinner posterior horn; flatter and more inwards facing posterior horn.
2		Smaller posterior horn with longer anterior horn; overall smaller meniscus with shorter anterior horn.
3		Thinner posterior horn with slightly longer and wider anterior horn; slightly smaller meniscus with shorter anterior and posterior horn.
4		Wider posterior horn; smaller posterior horn.
5		Slightly wider posterior horn with wider main body; slightly smaller posterior horn and smaller main body.
6		Overall smaller arch with wider anterior horn; overall wider arch with thinner anterior horn.
7		Overall wider arch with smaller posterior horn; overall smaller arch with wider posterior horn.
8		Overall smaller arch with smaller anterior and posterior horn; overall wider arch with wider anterior and posterior horn.
9		Slightly more posterior facing and thicker posterior horn; slightly more anterior facing and thinner posterior horn.
10		Wider posterior horn and body; smaller posterior horn and body.

This extended data figure presents two visual tables showcasing the top 10 principal component modes for the shape variation in the Lateral and Medial Meniscus. For each meniscus type, 3D models depict the morphological changes across the patient population at -3 standard deviations (left), the mean shape (center), and +3 standard deviations (right). Expert radiological interpretations, provided for both the negative and positive standard deviation extremes, elucidate the anatomical significance of each deviation from the mean; interpretations for -3 standard deviations precede those for +3 standard deviations, separated by a semicolon. This side-by-side analysis allows for a detailed comparison of the shape variations in the knee's meniscal structures. Please note that for interpretive accuracy, the 3D models are oriented such that the Posterior aspect is aligned with the top of the page, and the Anterior aspect is directed toward the bottom of the page.

Back to Table of Contents

Supplementary Figure 6: Differential Cartilage Thickness in Femur Tissue Across Principal Component Extremes.

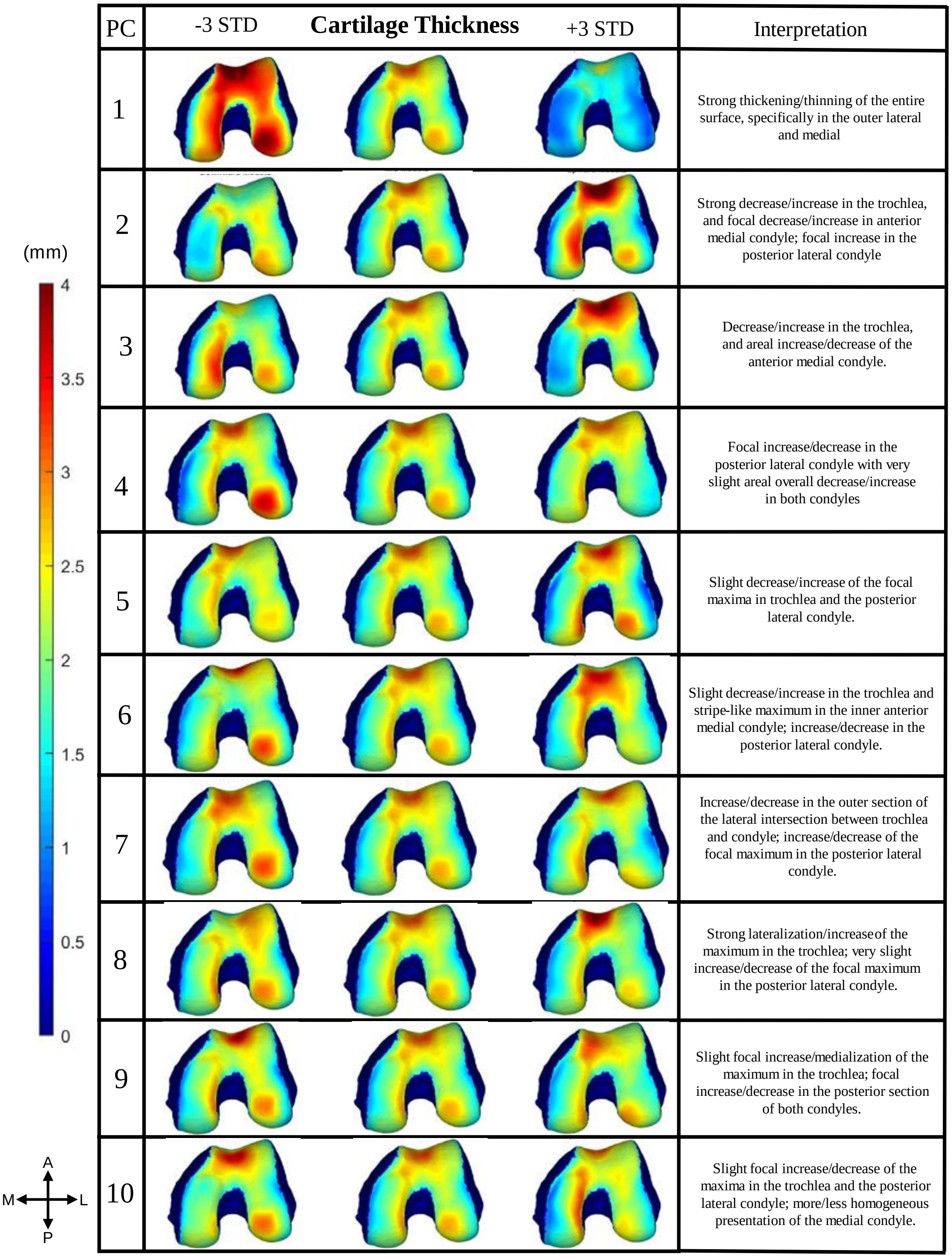

This extended data figure delineates the spectrum of femur cartilage thickness (mm) variations captured through the top 10 principal components (PCs). The visualization for each PC mode includes 3D representations for -3 standard deviations (left), mean cartilage thickness (center), and +3 standard deviations (right). Descriptions adjacent to each model articulate the deviations from the mean, with the terms to the left of the slash ("/") characterizing the -3 standard deviation state and those to the right detailing changes at +3 standard deviations. This structure of interpretation offers an immediate comparative view of the potential thickening and thinning across the range of standard deviations within the patient cohort.

Back to Table of Contents

Supplementary Figure 7: Principal Component Analysis of Patella Cartilage Thickness Variation.

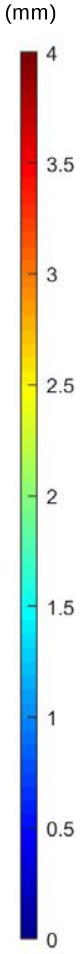

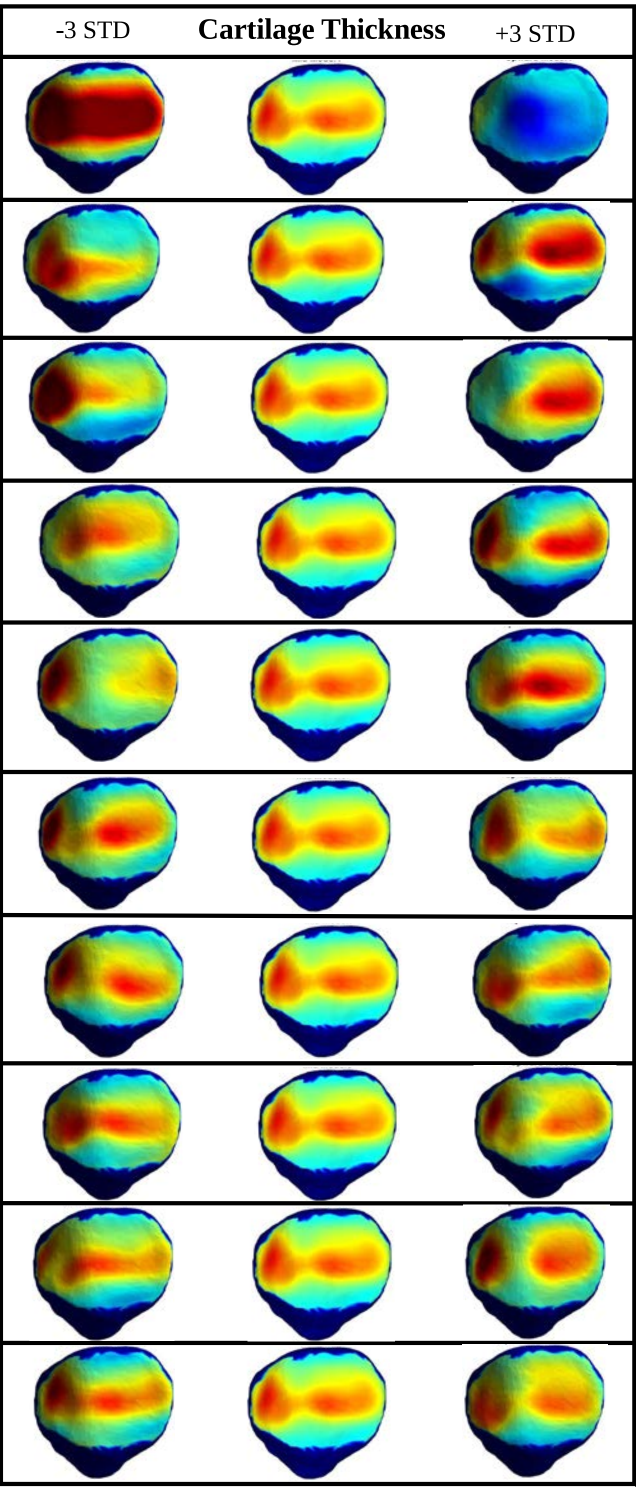

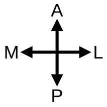

PC	Cartilage Thickness	Interpretation
1		Overall strong increase/increase with focus on the central region.
2		Strong decrease / increase of the maximum in the middle section of the lateral facets; increase / decrease in the inferior third with focus on the vertical ridge.
3		Areal decrease/increase of the lateral facet; areal increase/decrease of the medial facet.
4		Strong unification/ decrease of the maxima of the lateral and medial facet; increase/decrease in the outer region adjacent to the apex and base.
5		Lateralization/ Unified presentation of the maxima in the lateral facet to the maxima in the medial facet.
6		More focal presentation / slight lateralization of the maxima in the lateral facet and medial facet; increase/decrease in the medial facet.
7		Slightly more areal/ cranial and caudal presentation of the middle section of the lateral facet; slightly more focal/ caudal presentation in the areal maximum in the medial facet.
8		Shift of stripe-like maxima / more focal and medial presentation of the medial facet.
9		Less / more areal presentation of the maximum in the lateral facet; strong decrease/ slight increase of the maximum in the medial facet.
10		Less/ more areal presentation of the maximum in the lateral facet; increase / decrease of the stripe like maximum in the middle third of both facets.

Displayed in this extended data figure are the principal component (PC) modes that capture the variations in cartilage thickness of the Patella, with the scale in millimeters (mm). Each PC mode is visualized by 3D models at -3 standard deviations from the mean (left), the mean thickness (center), and +3 standard deviations (right). Accompanying the models, the descriptors to the left of the slash ("/") reflect changes at the -3 standard deviation end, while those to the right of the slash correspond to the +3 standard deviation end.

Back to Table of Contents

Supplementary Figure 8: Variability in Tibia Cartilage Thickness Through Principal Component Analysis.

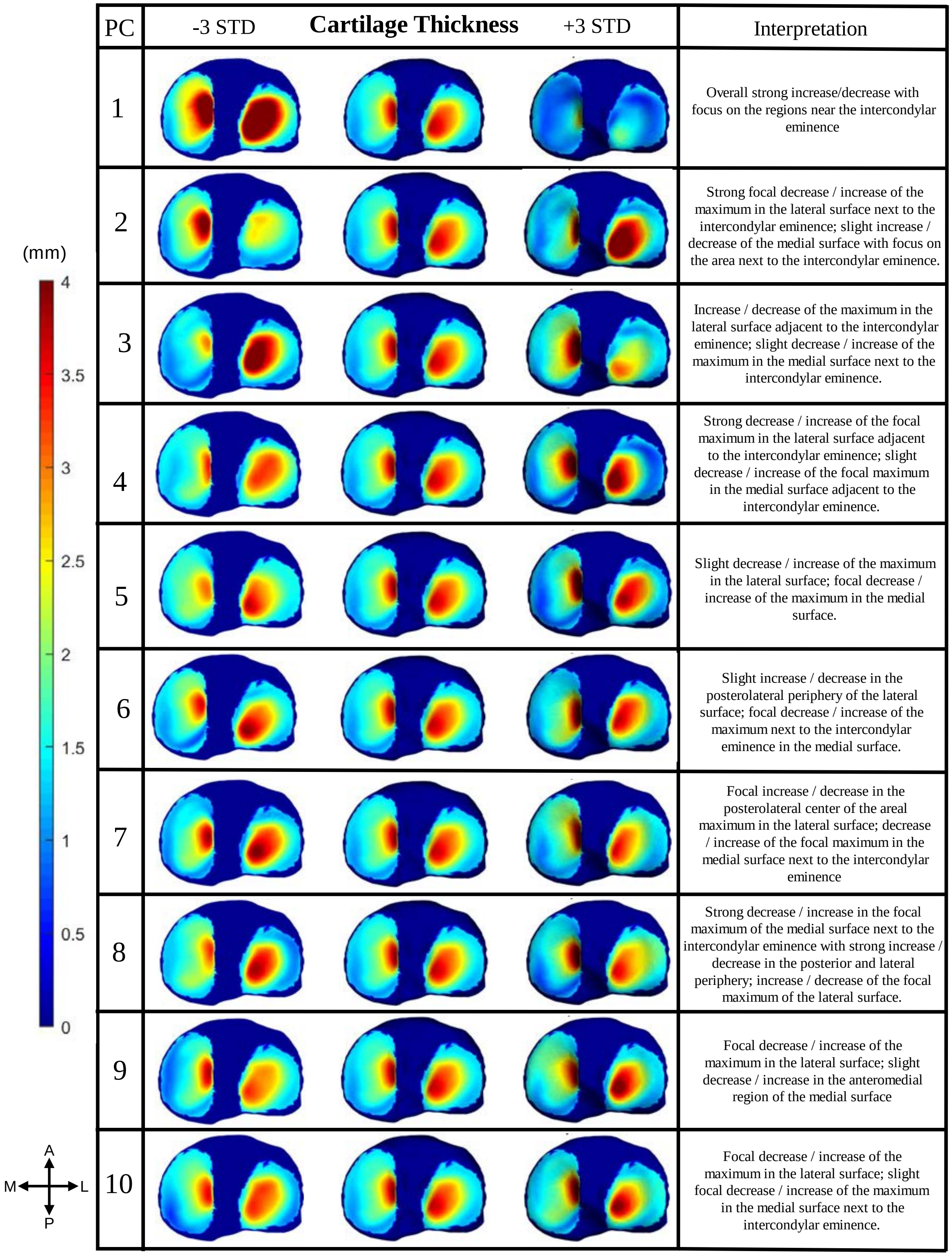

This extended data figure demonstrates the variability in Tibia cartilage thickness, quantified in millimeters (mm), as captured by the top 10 principal components (PCs). It showcases 3D models that represent variations at -3 standard deviations from the mean (left), the mean thickness (center), and +3 standard deviations (right) for each principal component. Annotations next to each model, divided by a slash ("/"), detail the observed changes: terms on the left describe the cartilage thickness at -3 standard deviations, and terms on the right for +3 standard deviations.

Back to Table of Contents

Supplementary Figure 9: Principal Component Analysis of Femur Cartilage T_2 Relaxation Time Variability.

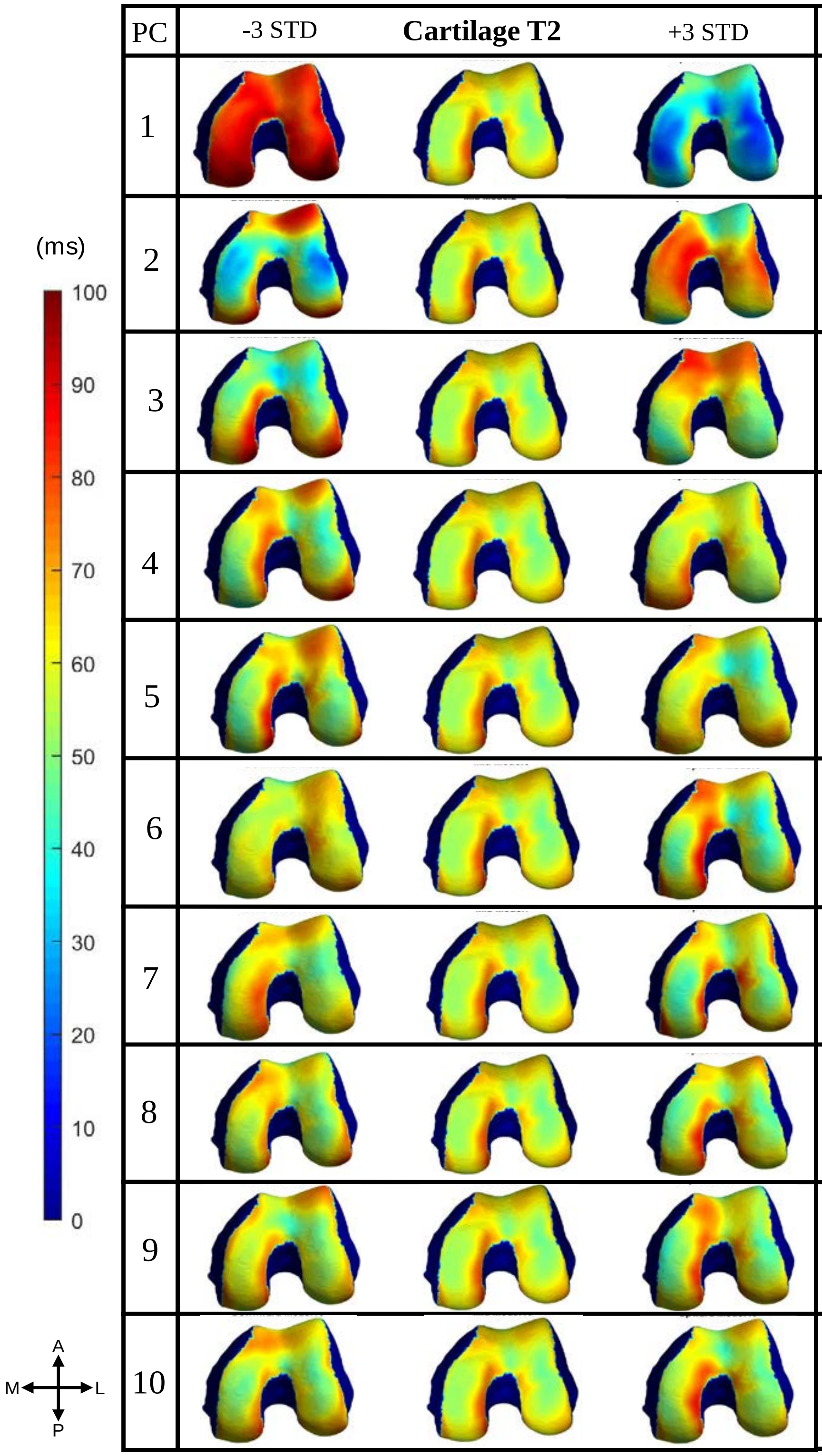

PC	Interpretation
1	Overall strong increase/decrease.
2	Strong areal increase/decrease in the lateral trochlea and the very posterior sections of both condyles and areal decrease/increase in the weight bearing regions on both sides.
3	Areal decrease/increase in the trochlea and areal increase/ decrease in the weight bearing regions.
4	Slight areal increase / decrease in the trochlea; increase / decrease in the posterior lateral condyle; decrease / increase in the posterior medial condyle.
5	Focal increase / decrease in the intersection of trochlea and lateral condyle; less / more homogeneous presentation of the condyles.
6	Focal decrease / increase of the upper medial trochlea; strong increase / decrease in the weight bearing regions and the very posterior lateral condyle.
7	Slight increase / decrease in the median trochlea; focal increase / decrease in the inner medial weight bearing region; slight focal decrease / increase in both very posterior condyles.
8	Slight focal decrease / increase in the very cranial region of the trochlea; slight increase / decrease in the lateral weight bearing area.
9	Focal decrease / increase in the medial trochlea; increase / decrease in the outer areas of both weight bearing regions.
10	Stripe like increase / decrease in the median trochlea; areal decrease / increase in the very posterior lateral condyle.

This extended data figure captures the top 10 principal components (PCs) reflecting the variability in Femur Cartilage T_2 Relaxation Time, presented in milliseconds (ms). For each PC, 3D models illustrate the variance at -3 standard deviations from the mean (left), the mean T_2 relaxation time (center), and +3 standard deviations (right). Beside each model, the descriptors separated by a slash ("/") offer insights into the changes: terms to the left pertain to the findings at -3 standard deviations, while terms on the right correspond to observations at +3 standard deviations.

Back to Table of Contents

Supplementary Figure 10: Variations in Patella Cartilage T_2 Relaxation Times Across Principal Components.

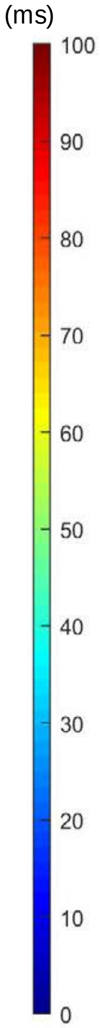

PC	-3 STD Cartilage T2 +3 STD	Interpretation
1	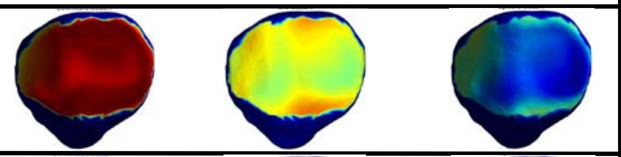	Overall strong increase/decrease.
2	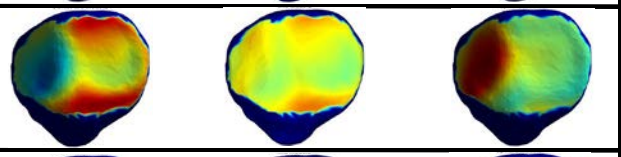	Areal decrease / increase of the medial facet and strong increase / decrease of the superior and inferior region of the lateral facet.
3	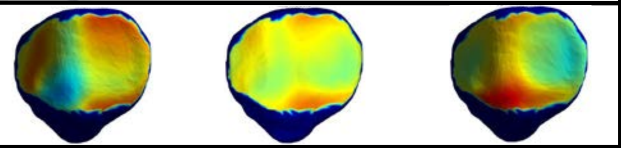	Decrease / increase in the inferior vertical ridge and strong increase / decrease of the medial facet and the superior region of the lateral facet.
4	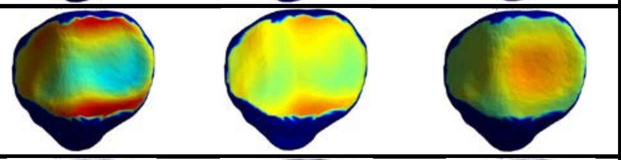	Strong decrease / increase in the central region of the lateral facet and slight decrease / increase in the superior and inferior region of the lateral facet as well as slight areal increase / decrease in the medial facet.
5	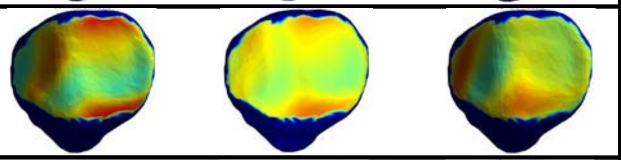	Focal increase / decrease in the superior vertical ridge and the inferior inner medial facet as well as decrease / increase in the outer superior region of the medial facet and more / less homogenous presentation of the lateral facet.
6	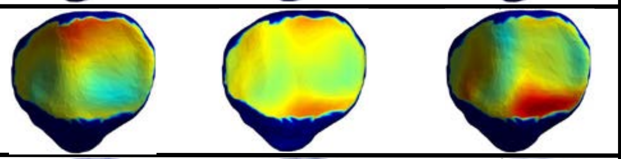	increase / decrease in the vertical ridge with focus to the superior section and the outer section of the lateral facet and areal decrease / increase of the medial facet as well as strong decrease / increase of the inferior region of the lateral facet.
7	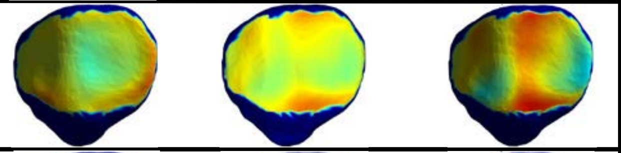	Strong decrease / increase in the lateral facet next to the vertical ridge and the superior medial facet as well as strong focal increase / decrease in the outer lateral facet and the inferior medial facet.
8	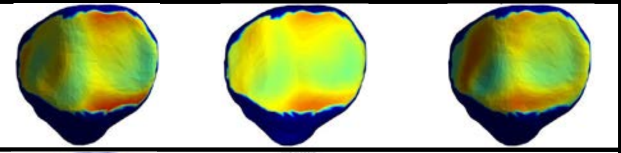	Slight increase / decrease in the central lateral facet and strong focal increase / decrease in the inferior inner region of the medial facet as well as decrease / increase in the superior outer medial facet.
9	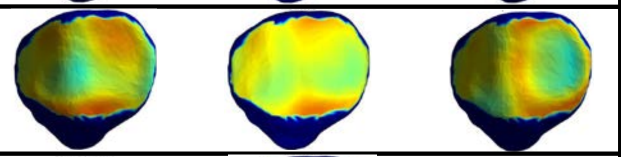	increase / decrease in the central lateral facet and decrease / increase next to the vertical ridge as well as focal increase / decrease in the inferior intersection between vertical ridge and medial facet.
10	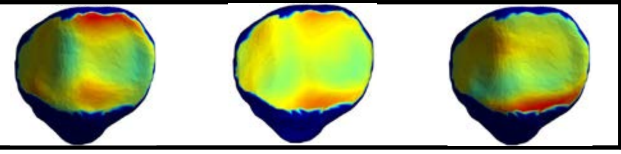	Strong decrease / increase in the very inferior region of the patella and stripe like increase / decrease in the inferior to middle section.

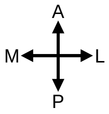

Illustrated in this figure are the principal component (PC) analyses revealing the range of T_2 relaxation times in Patella cartilage, expressed in milliseconds (ms). The figure delineates each of the top 10 PCs, showing 3D model variations at -3 standard deviations (left), mean T_2 times (center), and +3 standard deviations (right). Annotations provide dual interpretations for each standard deviation extreme, with terms before the slash ("/") relating to the -3 standard deviation scenario and those after the slash to the +3 standard deviation context.

Back to Table of Contents

Supplementary Figure 11: Principal Component Insights into Tibia Cartilage T_2 Relaxation Time Variability.

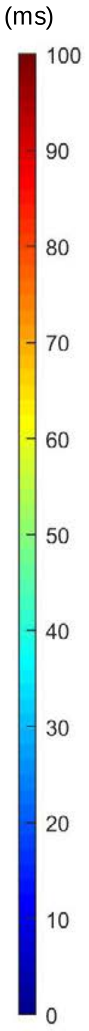

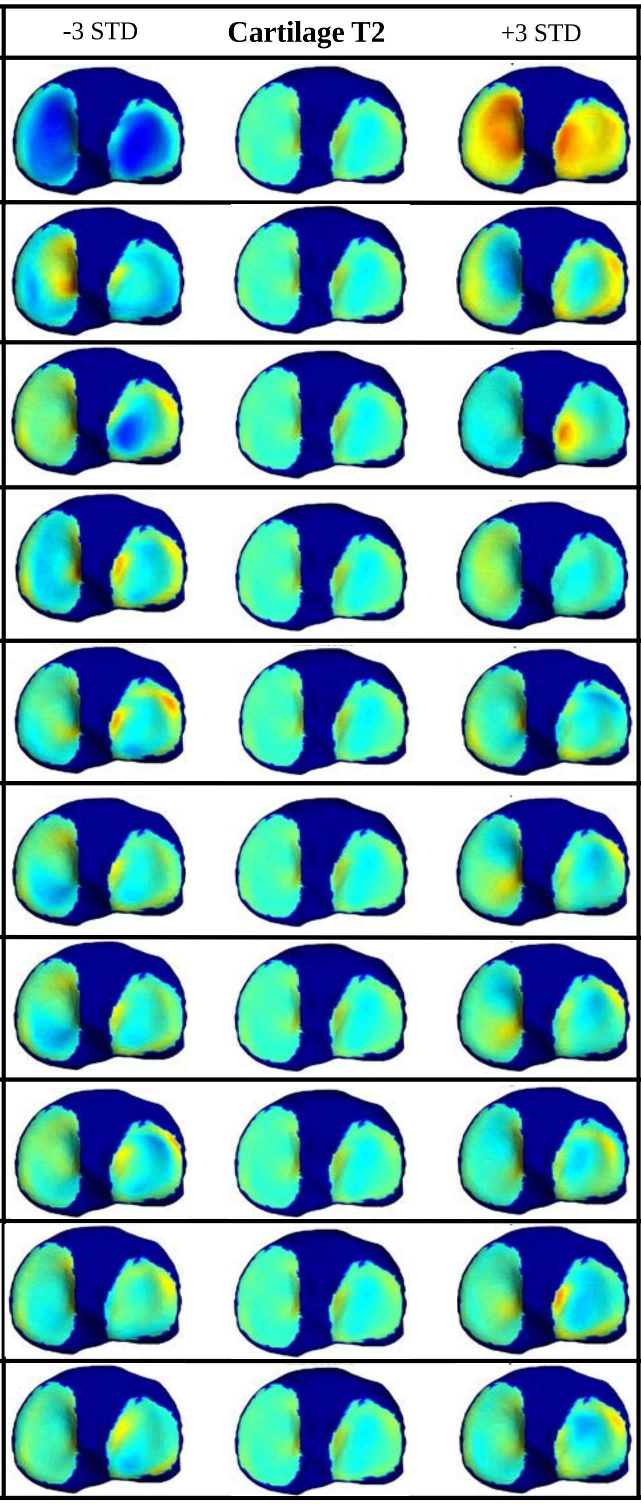

PC	Interpretation
1	Centrally pronounced but areal decrease / increase on medial and lateral joint surface.
2	Medial and lateral joint surface with focal central increase / decrease and peripheral decrease / increase.
3	Medial and lateral joint surface with focal central decrease / increase and peripheral increase / decrease, remaining surface with slight areal increase / decrease.
4	Slight central, areal decrease / increase on the medial facet, peripheral increase / decrease of both facets.
5	Decrease / increase in the posterior peripheral facets and focal strong decrease / increase next to the intercondylar tubercle in the medial facet.
6	Peripheral decrease / increase in the lateral and posterior region of the lateral facet; increase / decrease in the lateral periphery of the lateral facet.
7	Slight focal decrease / increase of the medial and lateral periphery of the lateral facet, Stripe-wise decrease / increase of the posterior part of the medial facet with slight areas increase / decrease of the anterior part.
8	Circular decrease / increase of the periphery of the lateral facet; slight areal increase / decrease of the medial facet.
9	Focal decrease / increase of the posterior and the medial periphery of the lateral facet with areal increase / decrease in the remaining surface.
10	Strong focal decrease / increase in the anterolateral and posteromedial periphery of the lateral facet; no notable change of the medial facet.

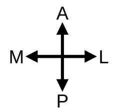

Captured via the analysis of the top 10 principal components (PCs), this figure showcases the variability in T_2 relaxation times within Tibia cartilage, measured in milliseconds (ms). Each principal component is represented by 3D models indicating the deviation at -3 standard deviations (left), the mean value (center), and +3 standard deviations (right). The dual interpretation next to each model, divided by a slash ("/"), allows for a nuanced understanding of changes: descriptors to the left refer to conditions at -3 standard deviations and to the right for +3 standard deviations.

Back to Table of Contents

Supplementary Figure 12: Residual Distribution of Meniscus and Bone Shape Biomarkers in Knee Replacement Analysis

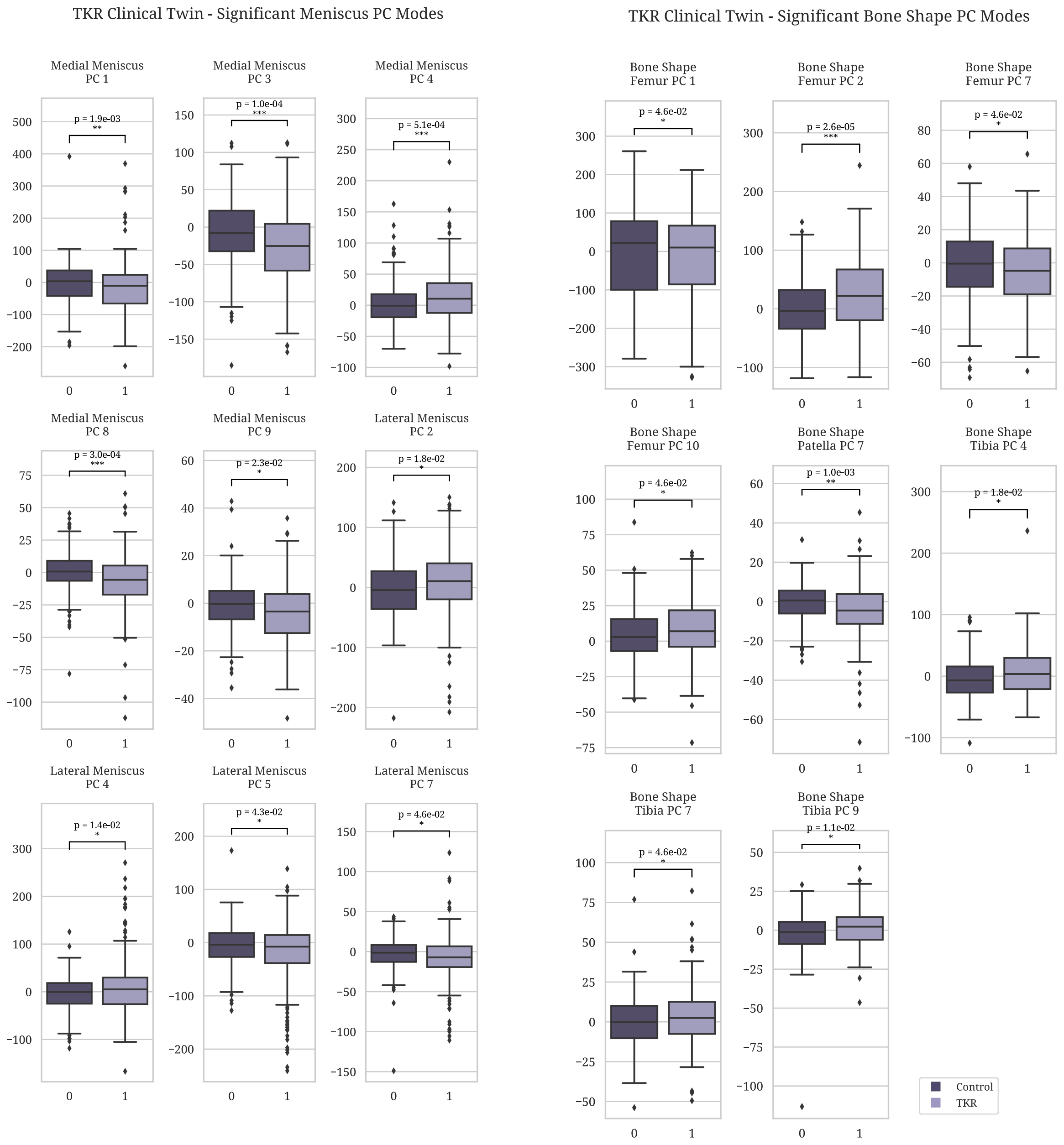

Box plots illustrate the distribution of significant principal component (PC) modes for bone shape and meniscus shape biomarkers between control and Knee Replacement groups. These features were identified using Paired Wilcoxon Rank Sum Tests, with Benjamini-Hochberg correction applied to address multiple comparisons. P-values, adjusted using the Hochberg method, are annotated above each plot. Significance levels are denoted as follows: single asterisk for p-values less than 0.05, double asterisks for p-values less than 0.01, and triple asterisks for p-values less than 0.001.

Back to Table of Contents

Supplementary Figure 13: Residual Distribution of Cartilage Thickness and T_2 Relaxation Biomarkers in Knee Replacement Analysis.

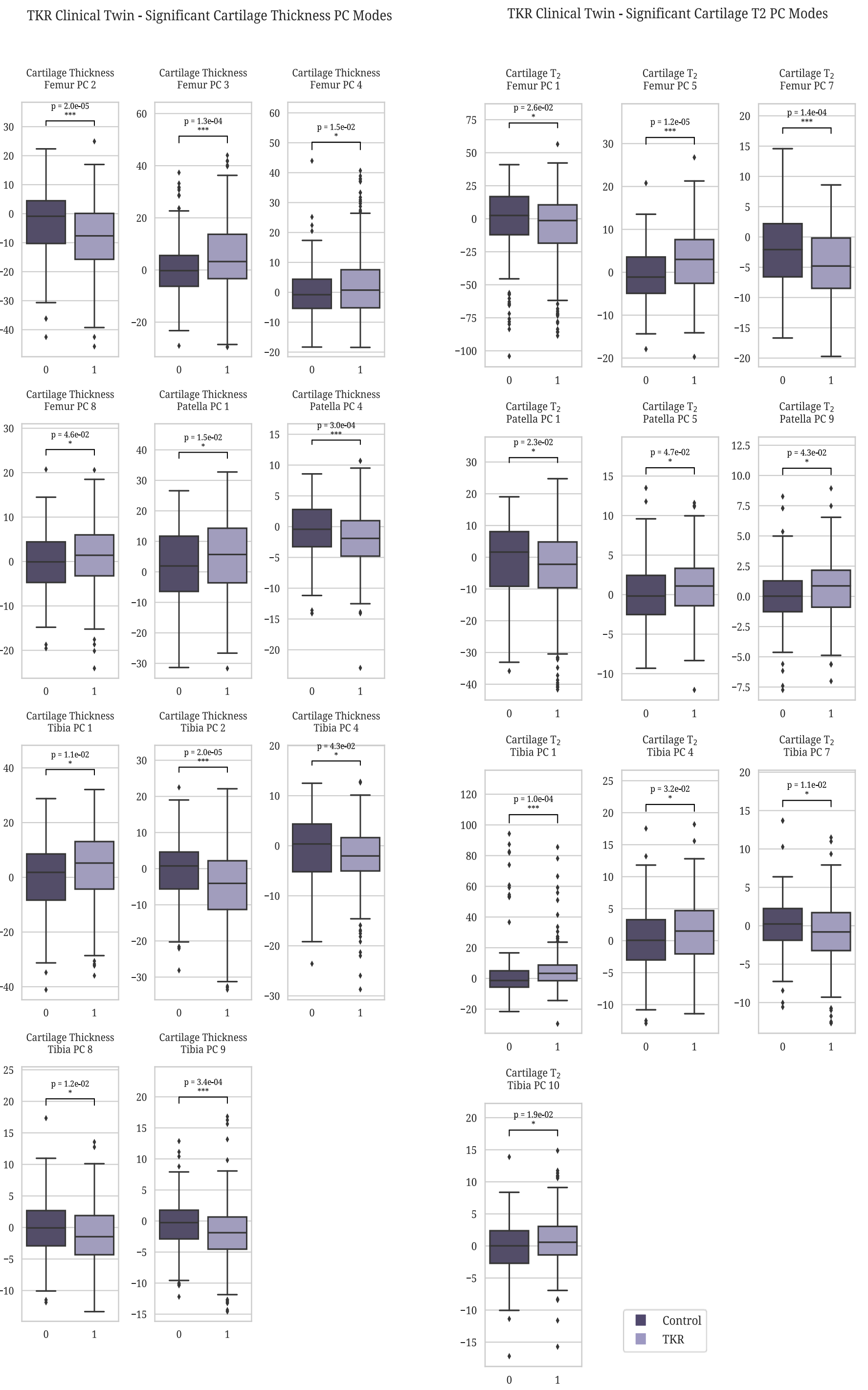

Box plots depict the distribution of significant principal component (PC) modes for cartilage thickness and cartilage T_2 relaxation time biomarkers between control and Knee Replacement groups. These features were identified using Paired Wilcoxon Rank Sum Tests, with Benjamini-Hochberg correction applied to address multiple comparisons. P-values, adjusted using the Hochberg method, are annotated above each plot. Significance levels are denoted as follows: single asterisk for p-values less than 0.05, double asterisks for p-values less than 0.01, and triple asterisks for p-values less than 0.001.

Back to Table of Contents

Supplementary Figure 14: Stability Selection of Imaging Biomarkers for OA Incidence and Knee Replacement Analyses.

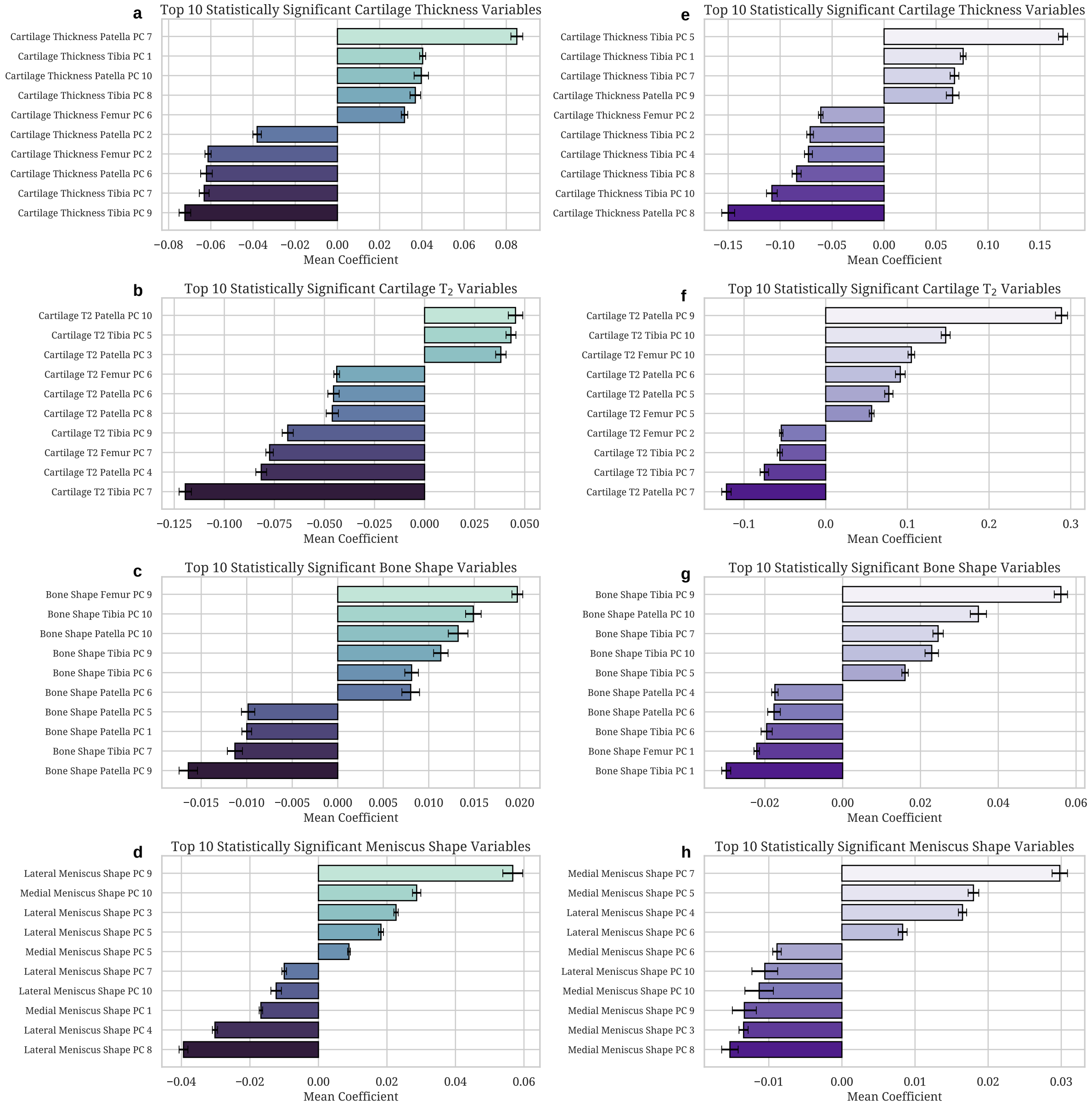

This figure presents the top 10 imaging biomarkers within each category (Cartilage Thickness, Cartilage T_2, Bone Shape, and Meniscus Shape) identified through stability selection for OA Incidence and Knee Replacement outcomes. **a-d) OA Incidence Stability Selection – Top 10 Features for Each Imaging Biomarker**: Bar charts for the top 10 significant variables in each imaging biomarker category (Cartilage Thickness, Cartilage T_2, Bone Shape, Meniscus Shape), ranked by mean coefficient magnitude. Error bars indicate 95% confidence intervals. **e-h) Knee Replacement Stability Selection – Top 10 Features for Each Imaging Biomarker**: Bar charts for the top 10 significant variables in each biomarker category, ranked by mean coefficient magnitude. Error bars represent 95% confidence intervals.

Back to Table of Contents

Supplementary Figure 15: Histogram Distributions of Key Features in OA Incidence and Control Groups.

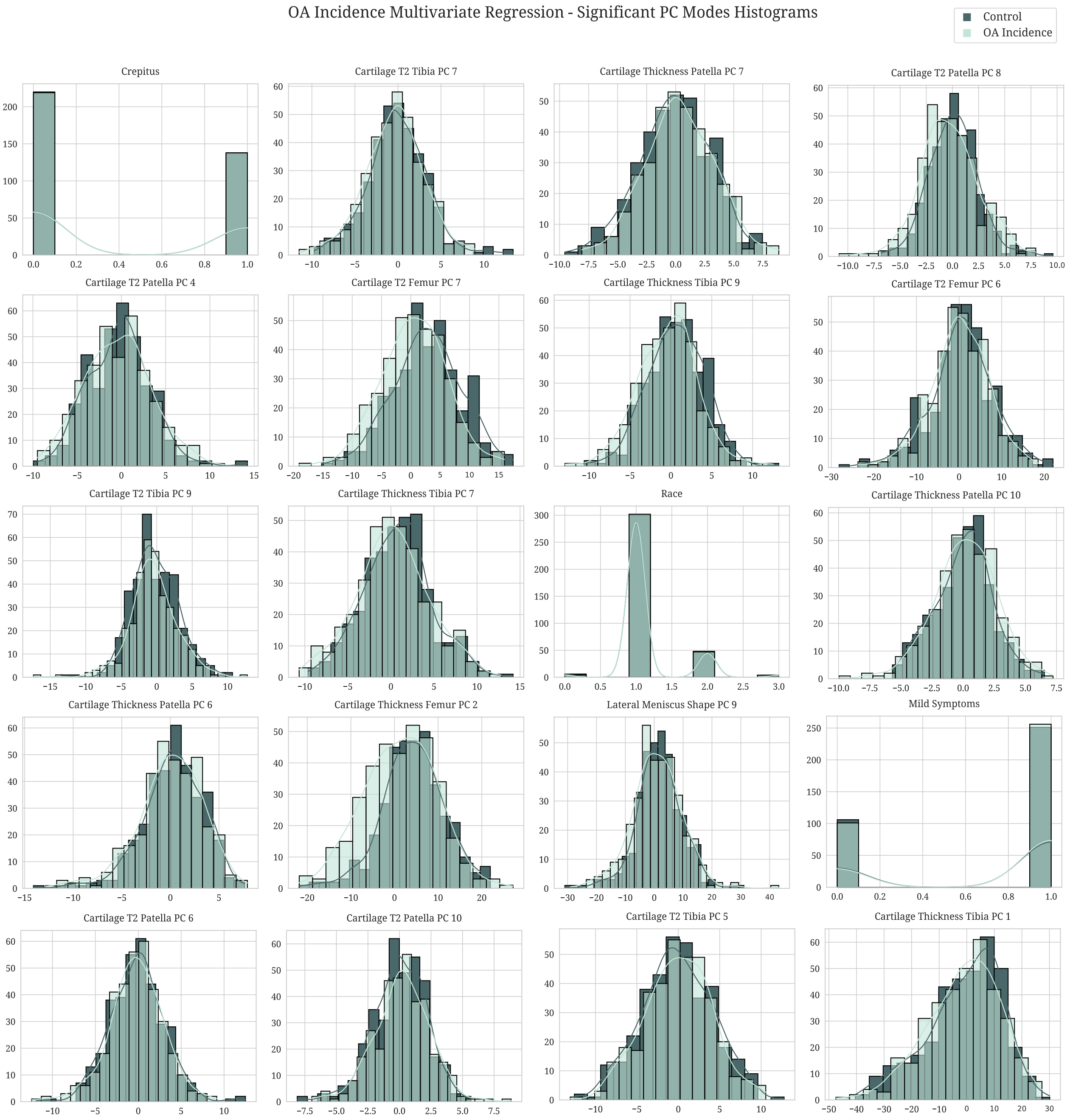

This figure shows the histogram distributions of imaging biomarkers identified as significant predictors of OA incidence through multivariate regression analysis. The plots compare key features between the OA incidence and control groups and reveal structural and compositional differences. Fitted density curves are included in each histogram to illustrate trends across the cohorts.

Back to Table of Contents

Supplementary Figure 16: Distribution Histograms of Key Predictive Features for Knee Replacement Risk.

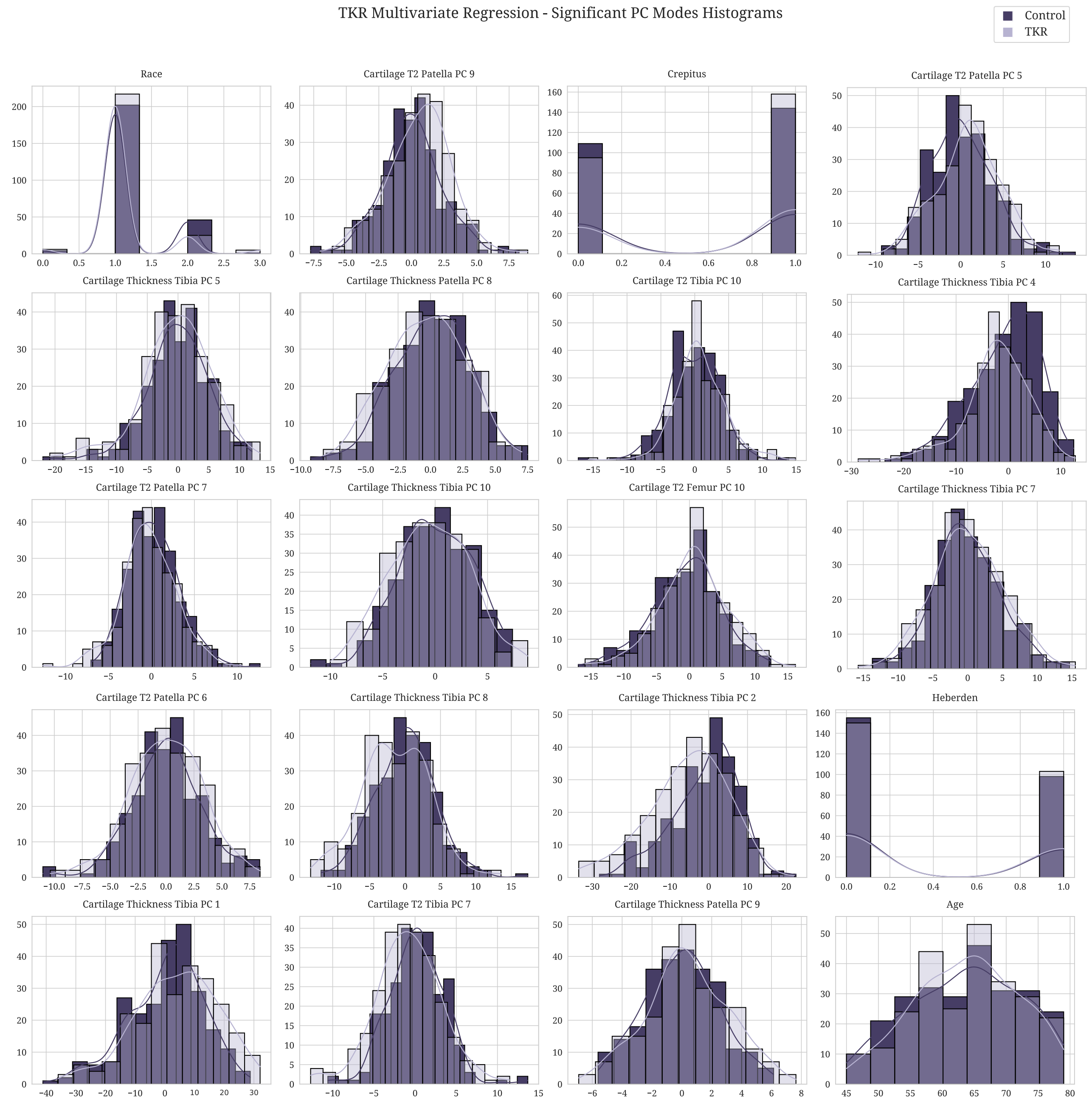

This figure displays histogram distributions of imaging biomarkers identified as significant predictors of knee replacement risk through multivariate regression analysis. The histograms and fitted density curves reveal differences in feature distributions between the control group and the knee replacement cohort.

Back to Table of Contents
